# Reliable Generation of Native-Like Decoys Limits Predictive Ability in Fragment-Based Protein Structure Prediction

**DOI:** 10.3390/biom9100612

**Published:** 2019-10-15

**Authors:** Shaun M. Kandathil, Mario Garza-Fabre, Julia Handl, Simon C. Lovell

**Affiliations:** 1 Division of Evolution and Genomic Sciences, School of Biological Sciences, Faculty of Biology, Medicine and Health, The University of Manchester, Manchester M13 9PL, UK; simon.lovell@manchester.ac.uk; 2 Cinvestav Unidad Tamaulipas, Km 5.5 Carretera Cd. Victoria-Soto La Marina, Cd. Victoria 87130, Mexico; mgarza@tamps.cinvestav.mx; 3 Decision and Cognitive Sciences Research Centre, Alliance Manchester Business School, The University of Manchester, Manchester M13 9PL, UK; julia.handl@manchester.ac.uk; 4 The Alan Turing Institute, British Library, 96 Euston Road, London NW1 2DB, UK

**Keywords:** protein structure prediction, fragment assembly, conformational sampling, stochastic ranking

## Abstract

Our previous work with fragment-assembly methods has demonstrated specific deficiencies in conformational sampling behaviour that, when addressed through improved sampling algorithms, can lead to more reliable prediction of tertiary protein structure when good fragments are available, and when score values can be relied upon to guide the search to the native basin. In this paper, we present preliminary investigations into two important questions arising from more difficult prediction problems. First, we investigated the extent to which native-like conformational states are generated during multiple runs of our search protocols. We determined that, in cases of difficult prediction, native-like decoys are rarely or never generated. Second, we developed a scheme for decoy retention that balances the objectives of retaining low-scoring structures and retaining conformationally diverse structures sampled during the course of the search. Our method succeeds at retaining more diverse sets of structures, and, for a few targets, more native-like solutions are retained as compared to our original, energy-based retention scheme. However, in general, we found that the rate at which native-like structural states are generated has a much stronger effect on eventual distributions of predictive accuracy in the decoy sets, as compared to the specific decoy retention strategy used. We found that our protocols show differences in their ability to access native-like states for some targets, and this may explain some of the differences in predictive performance seen between these methods. There appears to be an interaction between fragment sets and move operators, which influences the accessibility of native-like structures for given targets. Our results point to clear directions for further improvements in fragment-based methods, which are likely to enable higher accuracy predictions.

## 1. Introduction

In fragment-based protein structure prediction, short fragments of structural information are assembled into complete three-dimensional structures, typically using Monte Carlo strategies. These fragments are usually selected based on local sequence similarity to the target sequence, and are extracted from proteins with experimentally determined structures. To sample complete structures, fragment assembly protocols make use of heuristic optimisation procedures, whereby putative structures are selected or rejected by minimising the value of energy or scoring functions [1,2]. This is in line with the thermodynamic hypothesis of protein folding, which places the native structure at a minimum in the free energy landscape for a given protein. Since accurate descriptions of atomic interactions are computationally very expensive, most scoring or energy functions used in fragment-based methods are approximate or statistical in nature. Problems associated with inaccuracies in these scoring functions have been discussed in some studies [3,4]. Since structure prediction techniques work by finding local minima in these functions, significant disagreement with true physical energy landscapes can present challenges for structure prediction efforts.

**Figure 1:**
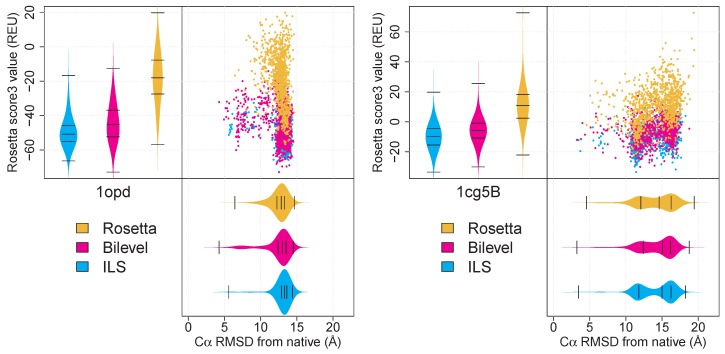
Examples of targets for which native-like states are rarely sampled (data from [5]). The top right panel in each set of plots is a scatterplot of Rosetta score values in Rosetta energy units (REU) versus Cα RMSD from the native structure. Kernel density beanplots for scores and RMSD values are shown next to the relevant axes. Data are shown for targets 1opd and 1cg5B, for Rosetta and our bilevel and ILS protocols (described in Section 1.2 and [5]), for 1000 low-resolution decoys each. It can be seen that the most frequently sampled states for these targets are found at a considerable distance from the native. The presence of a handful of decoys relatively near the native state indicates that these structures are compatible with the fragment sets used, and that it might be possible to increase the frequency with which these solutions are obtained.

In a previous study [5], we introduced two new sampling protocols designed to realise more extensive exploration of the available set of conformations within individual runs (a brief description of the methods appears in Section 1.2). We demonstrated that when good fragment sets are used, this improved exploration can translate into more frequent and reliable sampling of near-native basins in the energy landscape for many targets. While our results in cases of successful prediction would suggest that current scoring functions are sufficiently informative to guide the overall search, there are cases where the most frequently sampled structural states are substantially different from the native structure. Figure 1 shows some data for two targets on which such behaviour is seen. For these targets, a very small number of decoys are located fairly close to the native structure, hinting that the respective fragment sets can potentially be used to find a larger number of such structures. These observations raise two questions:

1. Is it simply the case that fragment set composition (interacting with the sampling algorithms) renders native-like states rarely accessible?
2. Is it the case that such structures were in fact sampled during many trajectories, but were not retained because of relatively unfavourable score values?

In [5], our experiments using older and newer fragment sets suggested that the fragment sets and choice of sampling algorithm do both play important roles. To address the first of the above questions, it is valuable to investigate the rate at which native-like local minima are accessed during runs of our protocols. The design of our bilevel and ILS protocols supports such investigations because they repeatedly descend into local optima in the search space, and a conformation is considered for retention as part of the final set of decoys if it is encountered as a local minimum (*LMin*) in the energy or score function landscape during the search. The analysis of encountered *LMin*s is in contrast to the approach normally taken, where only the energy or score value of the structures obtained at the end of the search is considered (e.g., [6,7]), and information about transiently accessed conformations is not readily available.

The second question arises from the fact that the size of the archive used to retain promising decoys is limiting, and that decisions about retention make use of only scoring information. In cases where good *LMin*s are accessed only rarely, it would be valuable to investigate whether and how relatively high-energy but otherwise promising structures can be retained, without fundamentally altering the behaviour of the search algorithms used. This issue necessitates the use of criteria other than score values to decide which structures should be retained during the search. Since score values do typically contain valuable information, such as the extent to which atomic clashes are avoided, it is desirable to combine scoring information with additional criteria, rather than to exclude scores completely. For targets displaying the behaviour shown in Figure 1, it can be seen that the most frequently sampled states are at considerable distances from the native structure. Assuming that native-like states are generated reasonably frequently during the search, it is possible that combining score functions with a second criterion employing a measure of conformational dissimilarity can improve the rate at which such structures are retained. A key idea here is that we continue to assume that the energy function is sufficiently informative, but we do not assume that the single global minimum in this function corresponds to the most accurate structure. Instead, we consider the multimodal nature of the energy landscape and try to identify a number of promising local minima, according to the above criteria. In this paper, we present a preliminary investigation of whether using such criteria when retaining putative decoys in runs of our protocols can result in improvements in the identification and retention of native-like decoys, without fundamentally altering the nature of the optimisation process itself.

The rest of this paper is structured as follows. The remainder of the Introduction contains a survey of alternative acceptance criteria for decoys sampled along trajectories of protein structure prediction methods, as well as a description of our sampling protocols originally introduced in [5], which formed the subject of this study. Having established the effectiveness of our protocols in comparison to the popular Rosetta method [8] in [5], we focused all our experiments presented in this paper on the two methods introduced in [5]. Section 2 then describes the results of our experiments, and Section 3 summarises the main findings and identifies opportunities for further development of our protocols. Finally, Section 4 provides details on the experimental procedures used.

### 1.1. Alternative Decoy Acceptance Criteria in Protein Structure Prediction

In this subsection, we consider and discuss work on the use of alternative criteria to accept putative conformations during protein structure prediction trajectories. We focus on techniques that do not rely on additional sources of information (e.g., experimental data or residue covariation-based constraints). Two key approaches are discussed below.

#### 1.1.1. Multiobjectivisation by Scoring Function Decomposition

Multiobjective optimisation problems are those that require the simultaneous optimisation of two or more objective functions [9]. These objectives are often conflicting, meaning that improving the value of one objective can lead to a deterioration in fitness measured by another objective, necessitating the use of specialised optimisation algorithms. Evolutionary optimisation techniques are commonly used, which arrive at solutions to an optimisation problem by considering a population of solutions. This population is subjected to various operations inspired by the biological process of evolution: existing solutions can be *mutated* or altered at random to produce new solutions, solutions can exchange parts of their decision variable sets in *recombination* operations, and solution quality is gradually improved by competitive *selection* of the fittest or most favourable solutions. Prominent examples are Non-dominated Sorting Genetic Algorithm-II (NSGA-II; [10]), Pareto Archived Evolution Strategy (PAES; [11]), and Strength Pareto Evolutionary Algorithm 2+ (SPEA-2+; [12]).

Single-objective problems can be recast as multiobjective ones, through a reformulation that retains the optimality of the original optimum solution(s) [13]. This process can sometimes produce optimisation problems that are significantly easier to solve [13,14]. For objective functions that are weighted sums, multiobjectivisation can be achieved through decomposition into two or more sets of terms. Alternatively, additional (helper) objectives can be introduced.

Since energy functions used in protein structure modelling are usually weighted sums of various energy terms, several methods have investigated the possibility of improving protein structure prediction by decomposing the energy function and casting the optimisation problem as a multiobjective one. For example, in [15], the CHARMM energy function was decomposed into two sets comprising bonded and non-bonded interactions, respectively. The authors demonstrated that this decomposition was useful in obtaining the correct structures for a few small proteins. This work was extended in [16], where it was shown that the same decomposition, when combined with more powerful search methods, could fold even larger proteins. Optimisation was carried out using a modified version of the PAES algorithm [17], and the authors reported improved predictive accuracy as compared to several approaches using a single-objective formulation. A further modification of this method [18] showed comparable results. The same energy function and decomposition was employed in [19] in conjunction with NSGA-II on a set of small proteins, with promising results. In [20], using the Amber99 energy function, the effects of a similar decomposition-based multiobjectivisation on search performance were investigated for a few simple algorithms. The authors stated that multiobjectivisation has the effect of removing or “smoothing out” many local optima in the original energy landscape, while retaining the guidance of the original energy function, which assigns lower energy values to more native-like solutions. In [21], a multiobjective evolutionary algorithm was employed with the Rosetta *score4* scoring function, which was decomposed into three sets of terms. The authors reported that the multiobjective algorithm is able to find conformations with lower scores as compared to standard Rosetta, which suggests an improved ability to escape local minima in the energy landscape. Comparisons against another evolutionary algorithm employing a single-objective formulation indicate that the multiobjective approach is able to locate a larger number of native-like solutions in several cases. Many other multiobjective formulations based around similar principles have been proposed (e.g., [22,23,24,25,26]).

#### 1.1.2. Diversity-Based Decoy Acceptance Criteria

In protein structure prediction, clustering by conformational similarity is a widely used method for identifying promising solutions after one obtains a large set of decoys (e.g., [27,28,29]). However, only a handful of methods appear to explicitly consider the structural diversity of generated conformations during the optimisation process. When deciding whether candidate conformations should be accepted during the search, measures of conformational dissimilarity are typically used as orthogonal acceptance or selection criteria in addition to score-based information. For example, a strategy employing dissimilarities between low-dimensional projections of candidate conformations sampled during fragment-based trajectories has been described [30]. Dissimilarity is computed between ultrafast shape recognition (USR) projections of the protein structures [31]. This dissimilarity information is used together with score information to select a subset of conformations at various stages of the search. These structures are then used to seed additional rounds of conformational sampling, and the authors found that this improves the algorithm’s ability to find diverse local minima in the energy landscape, occasionally with improved predictive accuracy. In [32], the authors proposed a method that successively employs two criteria for selecting a subset of structures from a larger ensemble of decoys sampled during fragment-based search (this is termed ensemble reduction). The first reduction is performed on the basis of energy or score values. The subset thus selected is further reduced on the basis of structural diversity, using an RMSD-based clustering approach that selects the centres of all clusters detected using a pre-defined clustering threshold.

Since evolutionary algorithms are typically population-based, the encouragement and retention of solution diversity is a widely studied and employed means of improving performance, especially when faced with multimodal objective or fitness functions [33]. A genetic algorithm has been described in [23,34], in which a parent individual in the population of structures is more likely to be replaced by a newly-generated child conformation if the parent is structurally similar to another parent structure. This scheme favours the retention of structurally novel decoys. In our own laboratory, we have described an evolutionary algorithm that uses a trade-off between Rosetta score values, and a low-dimensional measure of conformational diversity based on predicted secondary structure to inform ensemble reduction [35]. The diversity measure comprises root-mean-squared error based on a low-dimensional representation of the protein structure, defined in terms of the predicted start and end points of secondary structure elements. The trade-off between these two characteristics is achieved by adapting the technique of stochastic ranking [36]. Genetic operators were also used, which serve as an additional set of move operators, and the authors found that combining the stochastic ranking-based selection strategy with the use of genetic operators produced the most improvement in predictive accuracy distribution relative to Rosetta.

### 1.2. Bilevel and Iterated Local Search (ILS) Protocols

We recapitulate here some key features of our bilevel and ILS protocols (full details in [5]), which are the protocols studied in this paper. Our protocols are implemented as modifications of version 3.4 of Rosetta’s AbinitioRelax application [8]. A discussion of relevant details of the original AbinitioRelax method can also be found in [5]. Both protocols are built around a common framework:

1. Each run applies alternating rounds of perturbation and local search operators to the folding candidate structure. The perturbation steps are designed to encourage conformational exploration, and the operator comprises a single fragment insertion operation which is accepted regardless of its impact on energy or score value (which helps to escape from local minima). The local search operator performs fragment insertions and accepts new solutions greedily until 50 fragment insertions have been attempted without acceptance. At the end of each round of local search, the optimisation is said to have reached a local minimum (*LMin*).
2. A newly accessed *LMin* is compared to the *LMin* last encountered, and the Metropolis criterion [37] is used to define whether a new *LMin* should be accepted or rejected. The Metropolis temperature parameter is varied by a simulated annealing scheme.
3. An external archive maintains the set of the most favourable *LMin* structures over the course of each run, under the scoring function in use at any given time. All encountered *LMin*s are considered for addition to this archive. Once stored, the archived solutions are not used to inform the search process.
4. Our sampling strategies are active in Stages 2 and 3 of the Rosetta low-resolution protocol. Stage 1 can be seen as a randomisation/initialisation routine, and is used without modification. Following Stage 3, each structure retained in the archive is put through Stage 4 of the standard Rosetta low-resolution protocol.

The bilevel and ILS protocols differ in terms of how the perturbation and local search operators are composed. In the bilevel protocol, the perturbation operator only changes the conformational parameters of residues not predicted to be part of secondary structural elements according to PSIPRED [38] (the latter is run during the fragment generation step and does not constitute extra effort). The local search steps in the bilevel protocol affect only residues predicted to be in secondary structure elements. In this way, the decision variables (conformational parameters of each residue in the protein) are partitioned into two sets based on predicted secondary structure, and the resulting optimisation process follows a bilevel formulation [39,40]. The only difference between the bilevel protocol and the ILS protocol is that the ILS protocol is allowed to alter any part of the protein structure in both the perturbation and local search steps.

### 1.3. Outline and Contributions of This Study

In this work, we reformulate our bilevel and ILS methods to explicitly consider the multimodal nature of the problem by comparing alternative external archiving strategies for Point 3 in the framework described in Section 1.2. *LMin* structures encountered during the search are added to each archive, and the archives undergo an ensemble reduction step when a predefined maximum number of solutions has been stored. The mechanisms employed are based on those reported in [35], where stochastic ranking (SR) [36] is adapted as a means of achieving a trade-off between score values and conformational diversity. The SR method was originally proposed as a constraint-handling technique and shown to be effective in balancing the influence of objective and penalty functions, without resorting to (hard-to-tune) weighting factors. The work in [35] showed that SR could be adapted to balance the objectives of score and conformational diversity. We use SR as opposed to a fully multiobjective formulation (e.g., by scoring function decomposition), as we are not interested in extreme trade-off solutions, and SR gives us the ability to control the trade-off. To usefully answer the questions raised in the Introduction to this paper, it is essential that we retain the single-objective formulation of the problem. Building on the prior work [35], we make use of a different measure of conformational dissimilarity, namely the Hamming distance between binary contact maps [41]. The resulting archiver is denoted SRCM, reflecting the fact that the stochastic ranking procedure uses a structural dissimilarity measure based on contact maps. We incorporate this archiver in our bilevel and ILS protocols (described in [5]), and compare the performance of the new SR-based archiving strategy to our original energy-based strategy. A third archiving strategy, termed *elitist random* (ER), retains essentially a random selection of *LMin* structures (except one, which is retained based on energetic considerations), and thus serves as a baseline, allowing us to investigate the structures that would be retained without considering either score or conformational dissimilarity. The archivers differ only in terms of their ensemble reduction procedures; all encountered *LMin*s are added to all archives. Full details for the archiving strategies and their implementation within the bilevel and ILS protocols can be found in Section 4.

By direct examination of the decoys generated and retained by our protocols when using the various archivers, we investigate the relative importance of generation and retention of sampled structures in obtaining good predictive accuracy for a set of target proteins (Section 4.6). Although we focus on our bilevel and ILS protocols, the inferences we draw from our experiments are applicable to fragment-based protein structure prediction methods in general, and point to potential areas for improvement in our protocols as well as others.

## 2. Results

In this section, we describe the results of preliminary experiments assessing the ability of the SRCM and ER archivers to retain structurally diverse solutions in our bilevel and ILS protocols, as compared to the original energy-based strategy. Following this, we turn our attention to the rate at which native-like local minima (*LMin*s) are generated or accessed by runs of our protocols. We  then evaluate the effects of native-like *LMin* accessibility and choice of archiving strategy on distributions of predictive accuracy. Since our protocols and archivers are active in Stages 2 and 3 of the low-resolution protocol, we limit most of our analysis to structures sampled up to the end of Stage 3, however some results following Stage 4 are also discussed towards the end of this section.

### 2.1. The SRCM and ER Archivers Succeed in Retaining Structurally Diverse Conformations

By tracking the structures retained by the three archivers throughout multiple trajectories of each protocol, we verified that the SRCM and ER archivers are capable of retaining more structurally diverse decoys than the energy-based archiver. Detailed results and discussion of these evaluations can be found in the Supplementary Materials (Section S1). The ER archiving strategy typically retains the most structurally diverse decoy sets, followed by the SRCM and energy-based archivers. This reflects the fact that it retains a mostly random selection of *LMin* structures, which tend to be quite structurally diverse owing to the application of perturbation and local search steps in our search protocols. These results confirm that the SRCM and ER archivers can be used to evaluate the impact of inaccurate score values on decoy retention, as discussed in the Introduction, as they place importance on structural dissimilarity in addition to score values.

### 2.2. The Bilevel and ILS Protocols Access Native-Like Local Minima (*LMin*s) with Differing Frequencies

**Figure 2:**
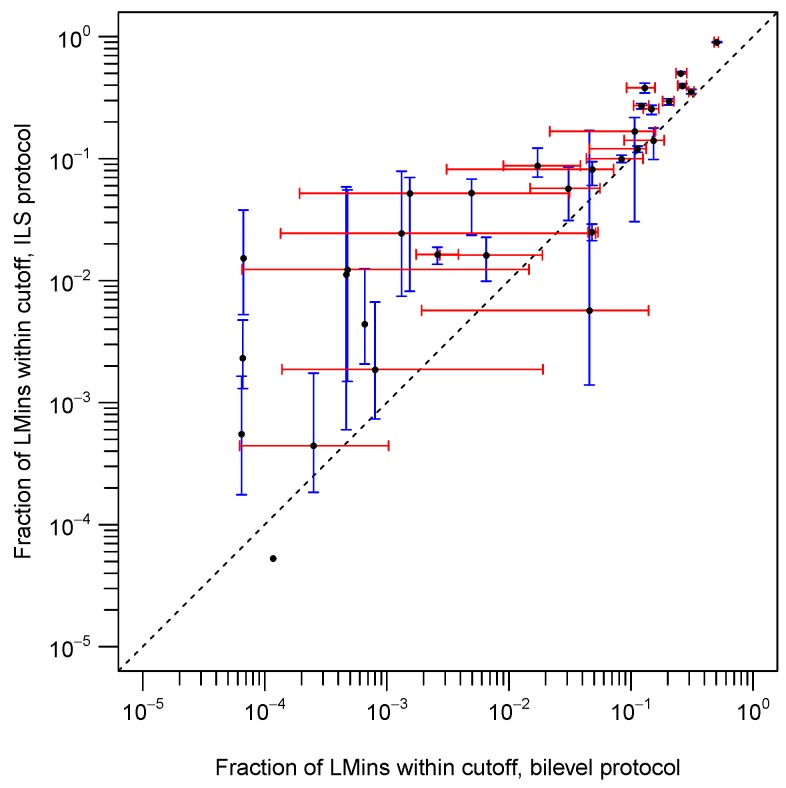
Log-log plot of the fractions of all accessed *LMin* structures that were within a Cα RMSD of 4 Å from the native structure, comparing the bilevel and ILS protocols. The ILS protocol typically accesses native-like *LMin*s at a higher rate than the bilevel protocol, although the opposite is true for a few targets. Median values (*n* = 100 runs) for targets in our test set are plotted as black points, and interquartile ranges (IQRs) are represented by error bars (bilevel IQR in red, ILS IQR in blue). An IQR is plotted only if the first quartile is non-zero, and medians are plotted only if values for both protocols are non-zero. A dashed line of unit slope is drawn to aid interpretation. An extended form of these data for all targets is given in the Supplementary Materials (Tables S3 and S4).

As mentioned in the Introduction, the rate at which native-like *LMin*s are accessed within runs of our protocols plays an important role in determining the distribution of predictive accuracies seen for a set of predictions for a target protein. Figure 2 compares the bilevel and ILS protocols in terms of the fraction of all accessed *LMin*s that were within a Cα RMSD of 4 Å from the native structure. It can be seen that, for most targets, the ILS protocol accesses native-like *LMin*s at a higher rate than the bilevel protocol, sometimes showing a difference of an order of magnitude or more. When the set of all 59 target proteins is considered, we found that median fractions of accessed native-like *LMin*s were significantly higher for the ILS protocol than for the bilevel protocol (Mack–Skillings test, *p* = 0.00044). There are, however, some targets for which the bilevel protocol outperforms the ILS protocol (e.g., targets 1c9oA, 1ctf and 2ci2I). For a few other targets, neither of our protocols ever accesses a single native-like *LMin* in all 100 runs, corresponding to a total of approximately 1.5 million *LMin*s sampled in total for each target and protocol. These are some of the most difficult targets in our dataset (targets 1dhn, 1fkb, 1rnbA and 1tul). For any search protocol, including ours, an inability to access even a single native-like *LMin* presents a fundamental barrier to attaining useful predictions.

Interestingly, there are some targets for which the ILS protocol (but not the bilevel protocol) can access at least a few native-like *LMin*s, and vice versa. In these cases, the median fraction of native-like *LMin*s accessed often differs considerably. Table 1 lists targets for which the median fraction of native-like *LMin*s differs between the bilevel and ILS protocols by a factor of 2 or more. The key difference between the bilevel and ILS protocols lies in the type of move operators used; the bilevel protocol uses secondary structure-dependent moves, whereas the ILS protocol does not. This suggests that, for the targets in Table 1, move operators play a strong role in determining the rate at which good *LMin*s are sampled, and therefore the rate at which they are retained by the different archivers. Other factors, such as secondary structure prediction accuracy and the quality of the fragment sets used could also play an important role [5].

### 2.3. Effective Conformational Sampling Has a Greater Impact Than Choice of Archiver

**Figure 3:**
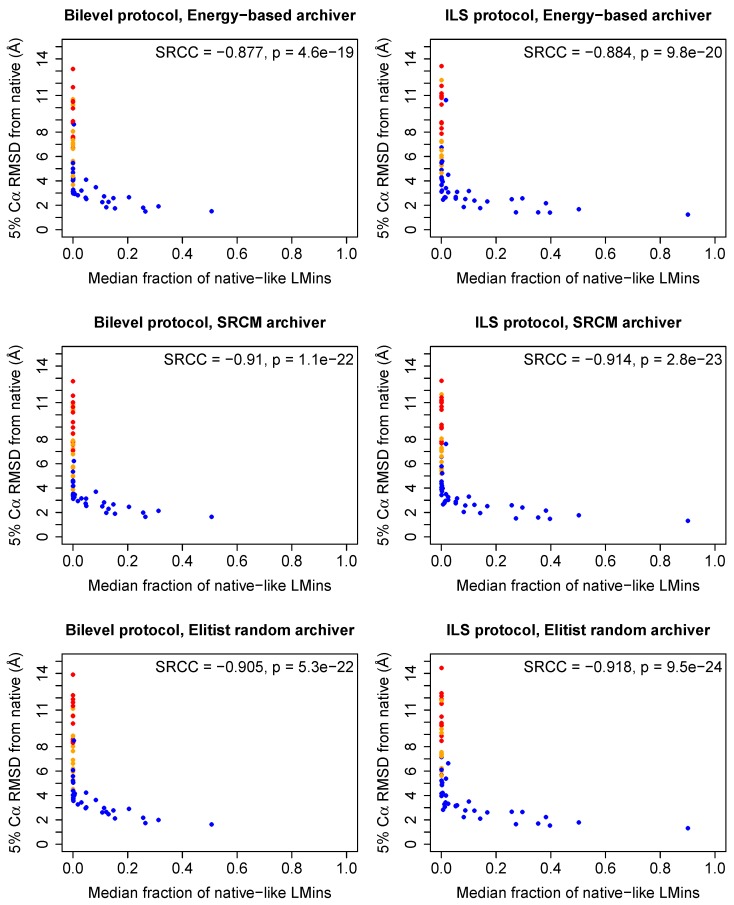
Accessibility of native-like *LMin*s correlates strongly with low-resolution predictive accuracy. *x*-axis: median fraction (*n* = 100 runs) of all accessed *LMin*s within individual runs that were within 4 Å Cα RMSD from the native. *y*-axis: 5th percentile Cα RMSD of all 1000 low-resolution decoys retained at the end of Stage 3. Each point represents data for a single target. Data are shown for all three archiving strategies, for runs of the bilevel and ILS protocols. Points in orange indicate targets for which the median fraction of *LMin*s within the specified RMSD cutoff is zero, and points in red indicate targets for which the *maximum* fraction of *LMin*s within the cutoff is zero, i.e., native-like *LMin*s are never accessed. All other points are marked blue. Predictive success is associated with reliable sampling of *LMin*s near the native structure. Spearman rank correlation coefficient (SRCC) values and approximate *p*-values are shown in each plot. *p*-values were corrected using the Bonferroni procedure.

The motivation behind trying to retain more diverse sets of structures is to determine whether this allows for the retention of structures normally discarded because of unfavourable score values, particularly in those cases where such states are native-like. Figure 3 shows the relationship between the median fraction of accessed *LMin*s that were within 4 Å Cα RMSD from the native structure, and eventual predictive accuracy in the decoy sets for each archiver and protocol (expressed as 5th percentile Cα RMSD from the native structure, following Stage 3 of the low-resolution protocol). Data for all targets can be found in tabular form in the Supplementary Materials (Tables S3 and S4), which also provide an idea of the dispersion in the fraction of native-like *LMin*s accessed across runs. A high median fraction of native-like *LMin*s indicates that a protocol is capable of reliably sampling *LMin*s near the native in many independent runs. This behaviour is associated with higher predictive ability, e.g., for targets 1aiu, 1elwA, 256bA and 5croA, for which both of our protocols show good predictive ability, indicating that the fragment sets for these targets readily permit access to near-native structures. Encouragingly, our protocols typically achieve favourable 5th percentile RMSD results even when the median fraction of native-like *LMin*s accessed is as low as 0.1, with all three archiving strategies. This indicates that on average, when good *LMin*s are reliably accessed by runs of our protocols, they tend to be identified and retained by all three archivers, up to Stage 3. As mentioned in Section 4.3 and Section 4.5, the SRCM and ER archivers employ elitism, meaning that one solution in each ensemble reduction step is always retained on the basis of score value. Thus, to an extent, all three archivers share any advantages that can be realised by retaining low-scoring solutions. For many targets, the low-resolution Rosetta scoring functions provide sufficient guidance to native-like states [5]. This could explain why 5th percentile RMSD values are typically comparable across the three archivers.

**Figure 4:**
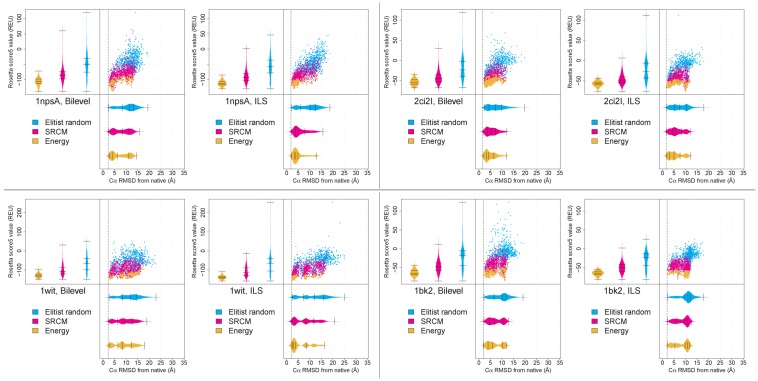
Score and RMSD data following Stage 3, comparing the bilevel and ILS protocols, each equipped with three archivers. Data are shown for four targets from Table 1. The left column shows data for two targets for which the ILS protocol accesses a higher fraction of native-like *LMin*s as compared to the bilevel protocol. The right column shows data for two targets showing the opposite trend. To the left and bottom of each scatterplot, kernel density beanplots are drawn for score and RMSD distributions, respectively. Lines on each bean indicate the five-number summary of each distribution. Each dataset is generated by 100 runs of the bilevel and ILS protocols, with each archiver retaining 10 structures per run. A dashed vertical line on each set of plots indicates the minimum RMSD from the native of any generated *LMin*, in all 100 runs.

Although there are no strong differences in 5th percentile RMSD among the three archivers, differences are much more pronounced when considering the full distribution of RMSD values. Figure 4 shows score and RMSD data (following Stage 3 of our protocols) for some of the targets in Table 1. The data in this figure also compare score and RMSD trends observed when using each of the three archiving strategies. Corresponding data for all targets can be found on Zenodo at https://zenodo.org/record/3356897. Our protocols can differ in the rate at which native-like *LMin*s are accessed (Section 2.2). It can be seen that, for any given archiver, a relative increase in the rate at which a given sampling protocol accessed native-like *LMin*s translates into a larger number of near-native decoys being retained. Due to the fact that the ER archiver keeps a (mostly) random sample of the *LMin*s encountered by the sampling protocol over the course of a run, the distribution of decoys obtained by this archiver represents the distribution of all *LMin*s, to some extent. Thus, an increase in the number of native-like solutions retained by this archiver between the bilevel and ILS protocols likely indicates that a larger number of such minima were encountered. Comparing the bilevel and ILS protocols, we see that a higher density of native-like decoys in the ER archiver associates with a corresponding increase in the density of such decoys in the other two archivers as well, and the SRCM and energy-based archivers tend to retain a greater number of native-like decoys.

Detailed analysis of the decoys retained by the three archivers (Supplementary Materials, Sections S2 and S3) shows that, although the SRCM archiver retains a greater number of native-like conformations than the ER archiver, the energy-based archiver often retains the largest number of such decoys. With a few exceptions (e.g., targets 1a32, 1lis, 1cg5B and 1opd), the SRCM and ER archivers typically do not retain a larger number of native-like decoys in cases where the energy-based archiver retains few or no such decoys. This suggests that one obtains no or only marginal benefit in terms of predictive accuracy when using the diversity-based archiving strategies (SRCM and ER), when considering the entire set of targets. As expected, the ER archiver retains decoys with higher scores, while the energy-based archiver retains decoys with the lowest scores among the three archivers (on average).

When the results for all protocols, targets and archivers are considered, it appears that, compared to the effect of using different archiving strategies, the frequency with which native-like *LMin*s are accessed has a stronger effect on the overall distributions of predictive accuracy in the decoy set. This could be due to a few reasons. The archivers operate only on *LMin*s, meaning that the distribution of conformational states in these structures defines what can be retained by any strategy operating on *LMin*s. The distribution of such states is determined by the interaction between the supplied fragment set and the conformational sampling method. We have already seen above that differences in move operators between our bilevel and ILS protocols can lead to changes in the rate at which native-like local minima are generated for some targets. These differences can have strong effects on predictive accuracy, and it is clear that if native-like *LMin*s are never accessed by the sampling protocol using the given fragment sets, they cannot be retained by any archiver operating on these structures. Further, as we have pointed out above, there are many targets in our test set for which the energy-based archiver already does a good job of retaining many native-like solutions. In such cases, the use of a criterion that rewards structurally distinct states can only be expected to produce relatively less favourable distributions of predictive accuracy, as compared to a purely energy-based retention scheme.

It may be possible to reduce the dependence of the archived sets on the distribution of all accessed *LMin*s by making use of the archived solutions to guide the search. As mentioned in point 3 in Section 1.2, the archived solutions currently do not influence the search in any way. The use of archived solutions to seed new rounds of conformational sampling is a common approach in evolutionary computation, and one that has been taken in other protein structure prediction methods (e.g., [30,32,35,42]). Iterative rounds of sampling beginning from structures deemed to be more favourable can allow the search to focus on more promising regions of conformational space. It may be valuable to investigate whether such schemes can improve robustness in our protocols.

### 2.4. Decoys Retained by Energy-Based Archiving Tend to Be of Higher Quality Following Stage 4 of the Low-Resolution Search

For completeness, we also investigated whether following further conformational sampling in Stage 4 (which uses three-residue fragments), decoys from the SRCM and ER archivers could exhibit more favourable RMSD values as compared to those from the energy-based archiver (details in the Supplementary Materials, Section S4). We found that the differences in predictive accuracy between the three decoy sets following Stage 4 were far less pronounced than at the end of Stage 3, and there were a few cases where the decoys retained by the SRCM and ER archivers produced a greater number of native-like decoys at the end of Stage 4, as compared to the energy-based archiver. Despite these successes, when all targets are considered, the decoys retained by the energy-based archiver contain a greater number of native-like decoys than the SRCM and ER archivers, on average. It is possible that results would change if Stage 4 were included as part of our sampling and archiving strategies, as this stage uses different fragment sets (and therefore move operators) and scoring functions.

## 3. Discussion

Difficult prediction scenarios for fragment-based protein structure prediction methods often associate with a reduced ability (or an inability) to sample native-like conformations. As pointed out in the Introduction, it is valuable to know whether ineffective sampling near the native state is a result of an inability to access or generate such structures using a given set of fragments and move operators, or whether scoring function inaccuracies lead to a failure to retain such conformations during the search. Our work in this paper suggests that the former is a primary limiting factor, and our results indicate areas for further development in our protocols.

In runs of our bilevel and ILS protocols, a direct analysis of all accessed local minima reveals that, in difficult prediction scenarios, for which neither of our protocols reaches native-like solutions, accessible local minima in the energy landscape are never found to be near the native. In other cases, such minima are difficult to locate reliably. This difficulty leads to a general inability to improve the frequency with which native-like structures are retained, employing criteria other than scores to retain good minima. In this study, we compared three schemes for retaining potentially promising structures encountered during runs of our bilevel and ILS protocols, and found that reliable access to native-like local minima had a stronger effect on distributions of predictive accuracy than the choice of retention strategy. While the SRCM and ER archives do succeed in retaining diverse sets of structures, this does not typically translate into an improved number of native-like decoys in the final set of predictions, as compared to a purely energy-based retention scheme. This points to the generation of good local minima being a primary limiting factor for successful prediction, rather than the rejection of such local minima based on inaccurate scoring information. This finding is also supported by our previous findings [5], where we saw that changing the fragment set used can have drastic effects on predictive accuracy. The strong effect of changing fragment library is likely to be due to one fragment set allowing much more frequent access to native-like structures, which in turn means that a greater proportion of the fragments in such a library correspond to the native state.

When our protocols are compared, we found that the bilevel and ILS protocols can differ considerably in the rate at which good local minima are accessed, and that this results in corresponding differences in distributions of predictive accuracy in the respective decoy sets. Within runs of a given protocol, we occasionally find small improvements in the number of native-like solutions obtained using the SRCM strategy, following Stage 3. This finding is in general agreement with results in [35], in which the use of a similar stochastic ranking-based decoy selection strategy was found to produce small improvements in predictive accuracy distributions. These improvements were more pronounced when their SR strategy was combined with genetic operators (additional move operators), suggesting that the interaction between fragment sets and move operators may be valuable to investigate, particularly for harder prediction problems. The use of move operators that are not limited to structural information present in the fragment sets (e.g., additional move operators in the QUARK method [43]) may present further advantages, particularly in cases where fragment quality is limiting for some parts of the target protein.

The experiments in this paper present some opportunities for further development of our sampling protocols, and the findings are applicable to fragment-based methods more broadly, since all fragment-based methods are still bound by their ability to generate high-accuracy decoys using a given fragment set. It may be valuable to investigate the development of new move operators or the use of alternative metaheuristics for the search. As mentioned in Section 2.4, extending our sampling and retention strategies to the entire search could be worth investigating. Additionally, the use of alternative archiving strategies, and using the archived solutions to guide the search process, may help in cases where native-like states are rarely accessed.

## 4. Materials and Methods

The methods presented in this paper were applied to two fragment-based sampling protocols introduced in [5], and which are briefly outlined in the Introduction (Section 1.2). These protocols, termed the bilevel and ILS protocol, both employ alternating rounds of perturbation moves (where a single fragment insertion is performed and accepted regardless of its impact on energy) and greedy local search steps. The difference between the two protocols lies in how the perturbation and local search operators are composed; the bilevel makes these moves based on predicted local secondary structure, whereas, in the ILS protocol, the two operators can alter any part of the structure. Both protocols are implemented as modifications to Rosetta AbinitioRelax [8], and employ many aspects of the original method [5].

### 4.1. Assessment of *LMin* Accessibility and Predictive Accuracy

In the bilevel and ILS protocols, the search is said to have reached an *LMin* when 50 consecutive fragment insertion attempts have been rejected in the greedy local search step (see Section 1.2 for brief details). A structure can only be retained during runs of our protocols if it is an *LMin* structure, and therefore, only *LMin*s can be returned as part of the final decoy set. Thus, the ability of the search procedure to access native-like *LMin* structures is valuable to investigate, since a good *LMin* structure can never be retained if it not accessed in the first place. For every *LMin* encountered in every run of our protocols, we recorded the Cα RMSD of this structure from the native, and we analysed the number and fraction of *LMin*s that were native-like, using a Cα RMSD cutoff of 4 Å from the native structure. The Mack–Skillings test [44,45] was used to assess differences in the median fractions of accessed *LMin*s between the two protocols, for all 59 target proteins. Significance was evaluated at the α=0.05 level.

For assessment of *LMin* data and eventual predictive accuracy, 100 runs of the bilevel and ILS protocols were run, and decoys were collected at the end of Stages 3 and 4. Score and RMSD data were compared using scatterplots and kernel density beanplots, as implemented in the beanplot package [46] for R version 3.3.0 [47]. A Gaussian kernel was used for all beanplots. RMSD beanplots used a bandwidth of 0.7 Å, and score beanplots used a bandwidth of 5 Rosetta energy units (REU).

### 4.2. Stochastic Ranking-Based Archiving Strategy

In stochastic ranking [36], a bubble sort-like procedure is used to compare consecutive pairs of solutions in a list of such solutions. Following many sweeps of this procedure over the list of all solutions, a subset of the top-ranked solutions are then selected and returned as the selected subset. Thus, the stochastic ranking procedure defines an ensemble reduction process, in which an initial set of conformations is reduced to its most highly favourable subset, under the SR criteria. The characteristic feature of SR is the use of a single user-defined parameter that controls the probability of using the objective or the constraint penalty function for comparing two solutions at any point in the procedure. This eliminates the need to tune problem-specific weighting parameters, and allows the user to control the trade-off between the use of the objective function and the constraint when selecting a subset of solutions.

Here, we use a generalisation of the original procedure, to consider two different ranking criteria, rather than an objective and a constraint, as described by Garza-Fabre et al. [35]. As pointed out by these authors, the procedure can even be extended to consider more than two criteria. The ranking procedure, illustrated in Algorithm 1, operates on an initial list of *M* solutions, and returns a version of this list which has been sorted using SR. A subset of the top-ranked solutions can then be selected, which reflects the best subset of solutions under both ranking criteria. Two ranking criteria are used ($c_{1}$ and $c_{2}$), which, without loss of generality, are assumed to be minimised. The core of the ranking process is detailed on Lines 3–22 in Algorithm 1. This process is repeated a maximum of *I* times, similar to the approach taken in the standard bubble sort algorithm, following which the list of solutions is said to be ranked. We refer to a single application of this core procedure as a sweep. In each sweep, every adjacent pair of solutions in the list ($H_{j}^{*}$ and $H_{j+1}^{*}$) is compared, based on the two criteria $c_{1}$ and $c_{2}$, and their positions are swapped based on the ranking criteria. A swap occurs if the solution with the lower rank shows a better value in either criterion while being equally good in the other (Lines 5–8 in Algorithm 1). If the two solutions are equally favourable under both criteria, they are swapped with a probability of 0.5 (Lines 9 and 10).

**Algorithm 1** Generalised stochastic ranking-based procedure.**Require:** Solution list ($H\leftarrow \langle x_{1},x_{2},\cdots ,x_{M}\rangle$), ranking criteria ($c_{1},c_{2}$), bias parameter (ρ)
**Ensure:** Ranked list of solutions ($H^{*}$)1:$H^{*}\leftarrow H$2:**for**i←1**to***I***do**3:   **for** j←1 **to** M−1 **do**4:      choose *r* uniformly at random in range [0,1]5:      **if** $c_{1}\left(H_{j}^{*}\right)=c_{1}\left(H_{j+1}^{*}\right)$ **and** $c_{2}\left(H_{j}^{*}\right)>c_{2}\left(H_{j+1}^{*}\right)$ **then**6:         swap $H_{j}^{*}$ and $H_{j+1}^{*}$7:      **else if** $c_{1}\left(H_{j}^{*}\right)>c_{1}\left(H_{j+1}^{*}\right)$ **and** $c_{2}\left(H_{j}^{*}\right)=c_{2}\left(H_{j+1}^{*}\right)$ **then**8:         swap $H_{j}^{*}$ and $H_{j+1}^{*}$9:      **else if** $c_{1}\left(H_{j}^{*}\right)=c_{1}\left(H_{j+1}^{*}\right)$ **and** $c_{2}\left(H_{j}^{*}\right)=c_{2}\left(H_{j+1}^{*}\right)$ **and** r≤0.5 **then**10:         swap $H_{j}^{*}$ and $H_{j+1}^{*}$11:      **else**12:         **if** r≤ρ **then**13:            **if** $c_{1}\left(H_{j}^{*}\right)>c_{1}\left(H_{j+1}^{*}\right)$ **then**14:               swap $H_{j}^{*}$ and $H_{j+1}^{*}$15:            **end if**16:         **else**17:            **if** $c_{2}\left(H_{j}^{*}\right)>c_{2}\left(H_{j+1}^{*}\right)$ **then**18:               swap $H_{j}^{*}$ and $H_{j+1}^{*}$19:            **end if**20:         **end if**21:      **end if**22:   **end for**23:   **if** no swap operation occurred **then**24:      **break**25:   **end if**26:**end for**

Lines 12–20 capture the general procedure, where the solutions are compared based either on $c_{1}$ or $c_{2}$. Here, the choice of ranking criterion is made stochastically, based on the value of the user-supplied parameter ρ. The general procedure is also applied when one solution in a pair being compared is better in both ranking criteria. Thus, over a sufficiently large numbers of sweeps of the procedure, the parameter ρ controls the relative frequency with which $c_{1}$ and $c_{2}$ are chosen to rank solutions, effectively controlling a trade-off between the use of these criteria for ranking the solutions. In other words, a solution is more likely to reach the top of the ranked list if it is optimal in one or both objectives, subject to the setting of ρ. Lines 23–25 handle the case where the ranking is complete before the application of all *I* sweeps. In practice, setting I=M (where *M* is the number of solutions to be ranked) allows a sufficient maximum number of ranking sweeps. Further, values of *I* that are too high cause the ranking to be very strongly determined by the single criterion that is favoured by the setting of ρ [35,36].

### 4.3. Elitist Step and Choice of Ranking Criteria

Before the application of the ranking procedure described in Section 4.2, we select the structure with the lowest score under the current scoring function and move it to the top of the list of solutions. This is termed an elitist step. The solution moved to the top of the list is always selected as a member of the final, reduced subset of solutions. The elitist step ensures that, regardless of the setting of the parameter ρ, the current most energetically promising solution is always retained. Following the elitist step, the stochastic ranking procedure described above is performed on all the remaining solutions in the archive, forming the final ranked list.

The two objectives considered for the archiving procedure based on stochastic ranking are energy (low-resolution Rosetta score) and structural diversity. For a structure to be favourable under the energy criterion, its score must be lower than that of a competing structure. In other words, energy is a criterion that is minimised. As a measure of structural diversity, we use the Hamming distance between contact maps [41], although our implementation allows for any diversity measure to be used. Contact maps were computed using a Cα-Cα distance cutoff of 8 Å, as used in other work [5,41]. Since we want to encourage the sampling of diverse conformations, solutions are preferred if they improve the diversity of the retained set of structures (i.e., diversity as a criterion is to be maximised). To do this, we first calculate the pairwise Hamming distances between all the structures in the archive. We find and record the minimum distance of each structure to all other structures in the archive. Then, a solution has a higher rank according to the diversity criterion if its minimum distance to all other solutions being compared is greater than that of a competing solution.

### 4.4. Implementation within the Bilevel and ILS Protocols

In keeping with the approach taken for the score-based archiving strategy originally used, the proposed archiving strategy using stochastic ranking and contact maps (we denote this archiving procedure by SRCM from now on) operates on the *LMin*s encountered during the search. Since processing all *LMin*s encountered in any trajectory would be very computationally expensive, the archive ensemble reduction procedures need to be invoked periodically during the search. We defined a few rules governing these procedures, and other setups are possible.

*LMin*s are added to the archive until a specified maximum size is reached (we refer to this as max_size), following which we apply the ranking procedure and select a subset (base_size) of the top ranking structures for retention (ensemble reduction). base_size reflects the number of solutions to be retained following one application of the SRCM procedure, under most circumstances. A third parameter, desired_size, specifies the number of solutions desired at the end of the search. These parameters obey the relation desired_size ≤ base_size ≤ max_size.

When applying the SRCM procedure during the search, it is desirable to use a fairly large number of structures for the ranking procedure, even if desired_size is relatively small, since ranking a large number of solutions increases robustness. Estimations of fitness under the diversity criterion for every solution depend on all other solutions present in the archive, and more informed measurements of contribution to diversity in the retained set are possible with larger sets. The presence of stochastic elements in the ranking procedures also motivates the use of a moderately large sample of decoys for ranking.

In addition to these rules, we also force an ensemble reduction to desired_size whenever the scoring function changes during the search (e.g., between low-resolution stages, or between substages of Stage 3). This is because the (current) purpose of the archivers is to retain the desired_size best solutions under the current evaluation criteria, and the choice of the best solutions could change under new evaluation criteria. Further, since the archived solutions need to be re-evaluated under the new scoring function, reducing the archive to desired_size avoids the need to re-score a large number of solutions using the new scoring function. As done for the energy-based archiver in [5], each structure retained in the SRCM archive after the end of low-resolution Stage 3 is subjected to Stage 4 of the standard Rosetta low-resolution protocol.

We implemented a framework that allows multiple archivers to operate side-by-side within any run of our protocols, such that all archivers make decisions on exactly the same set of local minima sampled during the run. Whenever an *LMin* is encountered, it is added to each archive, and each archiver performs its ensemble reduction procedure if its size exceeds the specified maximum. This allows us to compare archiver performance effectively, even at the level of single runs.

### 4.5. Elitist Random (ER) Archiver

To further evaluate the effectiveness of the SRCM strategy, we also included a third archiving procedure, which we refer to as the Elitist Random (ER) archiver. The ensemble reduction step of this archiver retains the elitist step of the SRCM procedure described above, whereby one structure is chosen solely based on score. Further structures are chosen uniformly at random from the remaining set of saved *LMin*s, until the desired size is reached. The elitist step was included in the ER archiver because the primary objective of the conformational sampling protocol is the minimisation of score values, and therefore choosing at least one low-energy structure is desirable. Using the ER archiver, we can evaluate the effectiveness of the SRCM strategy relative to an archiver that essentially makes random decisions, completely ignoring score information (apart from the structure chosen by the elitist step).

### 4.6. Experimental Setup

Predictions were run on the same set of 59 targets used in [5], using the “newer” fragment sets used in that paper. Running parameters are summarised in Table 2. One hundred runs of the bilevel and ILS protocol were run for each target, with the increase_cycles parameter set to 100, as done previously. Each run was configured to use three archivers:

1. A purely energy-based archiver, as in [5];
2. a stochastic ranking-based archiver using contact maps (SRCM); and
3. an elitist random (ER) archiver.

To ensure no differences in the total number of ensemble reduction steps performed by each archive, all archivers were configured with desired_size set to 10, base_size set to 100, and max_size set to 200. base_size was set to half the value of max_size, which allows a set of solutions retained in one reduction step to be compared with an equal number of newly obtained solutions. This resulted in three decoy sets per protocol and target protein, each with a total of 1000 low-resolution decoys. For the SRCM archive, we fixed ρ=0.5, following the analysis by Garza-Fabre et al. [35], who showed that small deviations from this setting can introduce strong biasing effects favouring one ranking criterion over another. To compare the performance of these archivers, we made a few different assessments:

#### 4.6.1. Intra-Archive Diversity Assessment over the Course of Each Run

To evaluate the effect of the archivers on the diversity of each retained solution set, we considered the distribution of the pairwise structural dissimilarity between the structures in each archive. This provides a direct test of the archivers’ ability to maintain a diverse solution set as each run proceeds. As measures of structural diversity, we calculated the Hamming distance between the contact maps of the stored structures, as well as the pairwise Cα RMSD between the stored structures. We used two measures since Hamming distance is used in the decision-making process in the SRCM archiver, whereas RMSD is not. Pairwise dissimilarities within archived structures were calculated following each ensemble reduction step, allowing us to track the evolution of structural diversity over the course of the run. We expect that an archiver that is effective at retaining diverse solutions will maintain a high average dissimilarity between the structures in the archive over the course of a run. In contrast, an archiver that is less effective may display a decrease in average dissimilarity over the course of a run, meaning that by the end of the run, most of the stored solutions in the archive are highly similar. The structural diversity in the final decoy set output by each archiver was also assessed using pairwise Cα RMSD. The empirical cumulative distribution function (ECDF) was derived and plotted as a step function for each archived set of 10 structures (giving a total of 45 pairwise distance values), and 100 ECDFs for were obtained for each target and archiver.

#### 4.6.2. Number of Native-Like Solutions Retained

We assessed the number of solutions retained by each archiver (over 100 runs) that were within 4 Å Cα RMSD from the native structure for each target. This information was collected after Stage 3 and 4 of the low-resolution protocol. Data for the three archivers were compared using the Mack–Skillings test and the corresponding post-hoc procedure. Critical values of the test statistic for post-hoc procedures were calculated using Monte Carlo simulation (50,000 iterations) assuming an experiment-wise error rate of α=0.05. The “conservative” version of the post-hoc procedure was used (Comment 82 in [45]).

### 4.7. Code and Data Availability

The bilevel and ILS protocols are available at https://github.com/shaunmk/Bilevel-ILS-Rosetta as patches for Rosetta version 3.4. Instructions for enabling the archivers mentioned in this paper are provided in the Readme. The fragment sets used in this work are the “newer” fragment sets used in [5] and are available at: https://zenodo.org/record/1254031. Extended data from some of the experiments in this study can be found at: https://zenodo.org/record/3356897.

## Figures and Tables

**Table 1:** Median fractions of accessed *LMin*s that were within 4 Å Cα RMSD from the native structure, comparing the bilevel and ILS protocols. Data are shown for targets for which the bilevel and ILS protocols differ in median fraction by a factor of 2 or more (last column). Median fractions were calculated over 100 runs of each protocol.

Target	Median (ILS)	Median (Bilevel)	ILS:Bilevel Ratio
1a19A	0.02445	0.00132	18.52273
1bk2	0.00188	0.00080	2.34170
1bm8	0.00056	0.00000	N/A
1bq9A	0.01620	0.00651	2.48772
1c9oA	0.00005	0.00012	0.45392
1ctf	0.00000	0.00007	0
1ew4A	0.00007	0.00000	N/A
1fna	0.05202	0.00154	33.77922
1gvp	0.00005	0.00000	N/A
1ig5A	0.08739	0.01719	5.08377
1iibA	0.01129	0.00047	24.27957
1npsA	0.01531	0.00007	229.32894
1pgx	0.27250	0.12170	2.23911
1shfA	0.01236	0.00048	26.01558
1tif	0.00233	0.00007	35.28610
1tig	0.00012	0.00000	N/A
1urnA	0.00056	0.00006	8.60307
1utg	0.01643	0.00260	6.32653
1vcc	0.00442	0.00066	6.71324
1wit	0.05264	0.00495	10.63649
256bA	0.38180	0.12960	2.94599
2ci2I	0.00569	0.04567	0.12463
4ubpA	0.00247	0.00000	N/A

**Table 2:** Running parameters for all methods and archivers. The same set of parameters were used for both the bilevel and ILS protocols, and both protocols used the same set of three archivers.

***Archive Size Parameters***	
desired_size	10
base_size	100
max_size	200
***Running Parameters***	
increase_cycles	100
Number of runs	100
Total number of structures (low-resolution)	1000
ρ (SRCM archiver)	0.5

## Supplementary Materials

The following are available online at https://www.mdpi.com/2218-273X/9/10/612/s1.

## Abbreviations

The following abbreviations are used in this manuscript:

ILS	Iterated local search
*LMin*	Local minimum; a decoy structure at a local minimum in the energy landscape
SR	Stochastic ranking
SRCM	Stochastic ranking using contact maps
ER	Elitist random
RMSD	Root mean square deviation
ECDF	Empirical cumulative distribution function
