# Supplementary material for: Reliable Generation of Native-Like Decoys Limits Predictive Ability in Fragment-Based Protein Structure Prediction
Source: Biomolecules. 2019 Oct 15;9(10):612. doi: 10.3390/biom9100612 (PMC6843117; doi:10.3390/biom9100612)
Supplement: Supplementary file 1 [file biomolecules-09-00612-s001.pdf]

Supplementary Information for: Reliable generation of native-like decoys limits predictive ability in fragment-based protein structure prediction

Shaun M Kandathil, Mario Garza-Fabre, Julia Handl and Simon C Lovell

S1 Within runs, intra-archive diversity rapidly declines in energy-based archives, but not in SRCM or ER archives

Over the course of individual runs, we find that the SRCM and ER archivers are able to maintain sets of structurally diverse structures in the archive, as compared to the energy-based archiver. Figure S1 shows the evolution of pairwise diversity between the structures in each archive for single runs of the bilevel and ILS protocols on the target 1acf. It can be seen that as the run proceeds, the median pairwise dissimilarity between the structures retained following each reduction step decreases when using the energy-based archiver, particularly towards the end of stage 3. In comparison, the SRCM and ER archivers are better able to maintain diverse sets of structures. This is indicated by higher median values of pairwise Hamming distance and Cα RMSD between the archived structures maintained over the course of the run. The fact that the ER archiver maintains a high degree of structural diversity over the run indicates that the *LMin*s sampled during any trajectory are structurally diverse. This is due to the fact that besides the structure chosen by the elitist step in the ER ranking procedure, all other retained structures are a random subset of the *LMin* structures generated during a trajectory. As mentioned in Methods Section 4.3 in the main text, the use of the elitist step in the SRCM and ER archives ensures that some of the benefits of having accurate scoring functions are retained in these archiving strategies, as in the energy-based archiver. As a result, one retained structure is always common to all three archivers, up to the end of stage 3.

In Figure S1, a number of spikes can be seen in the median pairwise dissimilarity values in stage 3, for the energy-based and SRCM archives. These correspond to the reduction steps invoked when changing the score functions used in stage 3. As pointed out in Section 4.4 in the main text, these reduction steps reduce the size of the archive to the `desired_size` of 10 structures. Following these reduction steps, we observe that the energy-based archive retains a much less diverse set of structures than average, and these appear as sharp dips in the plots in the left column of Figure S1. In contrast, the equivalent reduction steps in the SRCM archiver retain more diverse sets of structures; the median dissimilarity in the retained set increases at these points in the run, appearing as upward spikes in values of Hamming distance-based diversity. This indicates that the

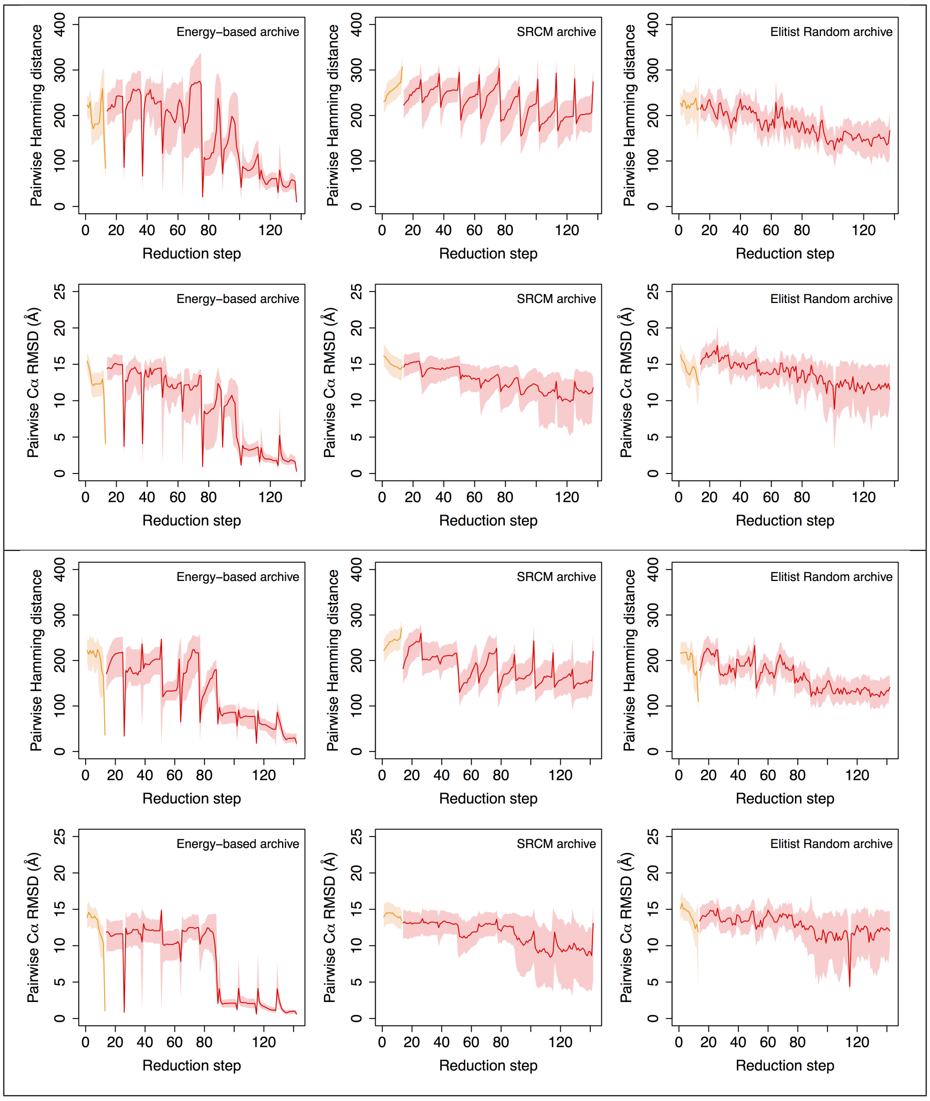

Figure S1: Intra-archive diversity over the course of a run, comparing three archivers. Data are shown for stages 2 and 3 (orange and red), for single runs of the bilevel and ILS protocols (top and bottom panels, respectively) for target 1acf. Median pairwise values of Hamming distance between contact maps (top row in each panel) of the archived structures and corresponding Cα RMSD values (bottom row) are plotted following each ensemble reduction step, for three archivers (left column: energy-based archive, centre column: stochastic ranking-based archive using contact maps (SRCM), right column: ER archive). Shaded regions represent the interquartile range of the pairwise dissimilarities at each reduction step. The SRCM and ER archivers maintain a higher degree of diversity in the retained set of structures, as compared to the energy-based archiver, and this is evidenced by higher median values of Hamming distance and Cα RMSD between the retained structures as the run proceeds. These trends are highly similar between independent runs for a given protein.

reduction of the SRCM archive to a small number of structures retains the very best structures in terms of both the energy and structural dissimilarity criteria. The very next reduction step (to `base_size` = 100 structures) typically displays reduced values of median pairwise dissimilarity, as a consequence of retaining a superset of structures that are not all as energetically favourable and structurally diverse as the top 10. We find that typically, of the 10 structures retained prior to each change in score function, the majority of these (usually 9 or 10) are retained even following the change in score function. This indicates that the observed drop in median diversity in the reduction step just after a change in scoring function is not a consequence of a loss of the top-ranking structures retained in the prior reduction step, but rather due to the relative reduction in median dissimilarity following the retention of a larger set of structures. We find that these behaviours are typical for all proteins tested.

Considering the diversity in the final set of structures retained at the end of stage 3 (Figure S2), we find that the energy-based archiver typically retains highly similar structures, indicated by a sharp increase in the cumulative distribution functions of pairwise RMSD at low RMSD values. The ER archiving strategy typically retains the most structurally diverse decoy sets, followed by the SRCM and energy-based archivers, which retain less diverse sets. The high structural similarity of solutions retained on the basis of only energy values suggests that in any given run, a singular basin in the conformational landscape is usually detected as the location of the structures with the lowest energy. In contrast, the SRCM and ER archivers appear to retain structures from distinct locations in the energy landscape.

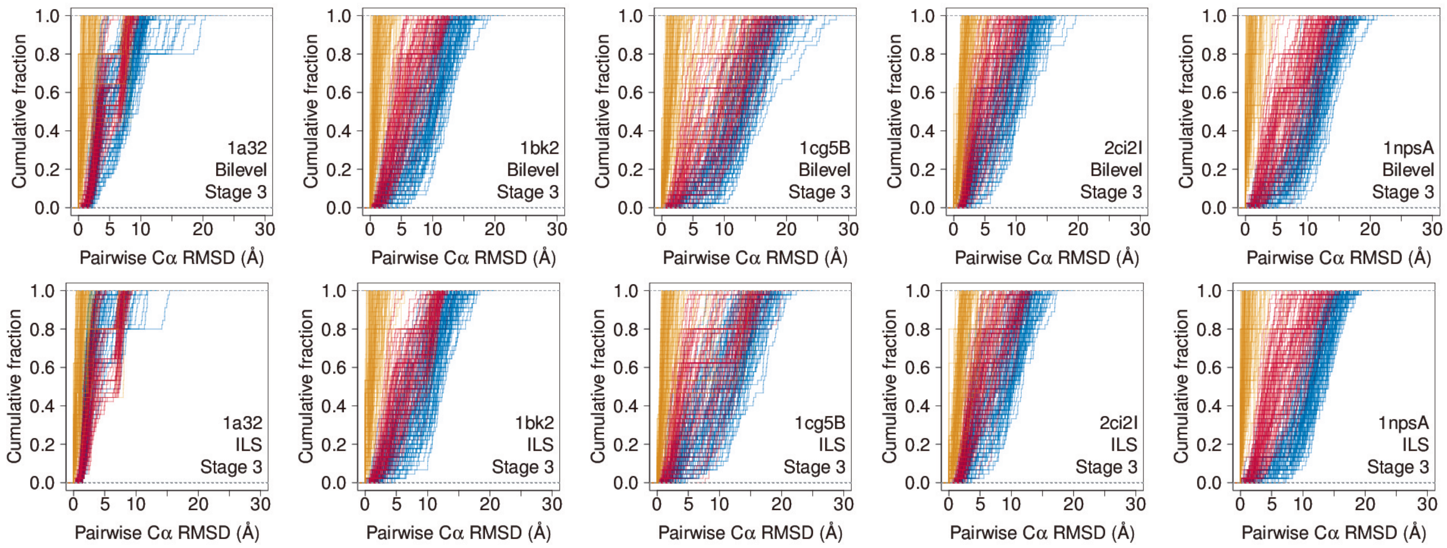

Figure S2: Empirical cumulative distribution functions (ECDFs) of pairwise Cα RMSD within energy-based (gold), SRCM (magenta) and ER (blue) archives. Results are shown for five targets (columns), following stage 3 of the bilevel and ILS protocols (top and bottom rows, respectively). Each line represents the ECDF of the distribution of pairwise RMSD values within a single archived set of 10 structures, following a single run of each protocol. There are 100 such ECDFs per target, protocol and archiver. Rapid increases in the ECDF at low values of pairwise RMSD indicate that the archived set of decoys is highly structurally similar.

S2 Analysis of low-RMSD decoy retention in the three archivers

Comparing the three archiving strategies across all targets (Figure S3), we find that both the energy-based and SRCM archiver retain significantly larger numbers of native-like structures in the final decoy set as compared to the ER archiver for a given sampling protocol (assessed as the number of decoys within 4 Å Cα RMSD from the native; Mack-Skillings test). This indicates that the use of selection criteria involving scores, and in the case of the SRCM strategy, diversity information, leads to better results than a primarily random selection strategy. Comparing the energy-based and SRCM archivers, however, we find that the energy-based archiver retains significantly larger numbers of native-like solutions across all the solutions.

Since our SRCM archiver employs a criterion based on structural dissimilarity, the relative frequency with which distinct conformational states are generated has an effect on what states will be retained. For many targets in our test set, our protocols show good predictive accuracy distributions even with the energy-based archiver, such that native-like states are frequently accessed and can be retained on the basis of energy alone. Indeed, we observe that the strongest differences between the archivers are seen on targets for which the energy-based archiver already retains a substantial number of decoys within 4 Å of the native. In these cases, it can be expected that the use of a diversity-based measure is more likely to favour the retention of states that are farther from the native, on account of their dissimilarity to the frequently accessed, native-like states. Such behaviour would result in the SRCM archives displaying less favourable distributions of predictive accuracy than the energy-based archives.

Considering targets for which the energy-based archiver retains few or zero structures close to the native, we find that typically, neither the SRCM nor the ER archiver is able to improve results. There are some exceptions to this rule (Table S1). For targets 1a32 and 1c9oA, we see that SRCM improves the number of near-native solutions following stage 3 relative to the energy-based archiver, in both protocols. Typically however, when the ER or SRCM archivers retain more native-like decoys, the increase in the number of such structures relative to the energy-based archiver tends to be quite small. Given the presence of probabilistic elements in the SRCM and ER archiving strategies, we cannot rule out the possibility that these smaller increases may be due to random effects. Nevertheless, within runs of a given protocol, the three archivers operate side-by-side, and decisions are made on the exact same set of *LMin*s, removing one source of variation.

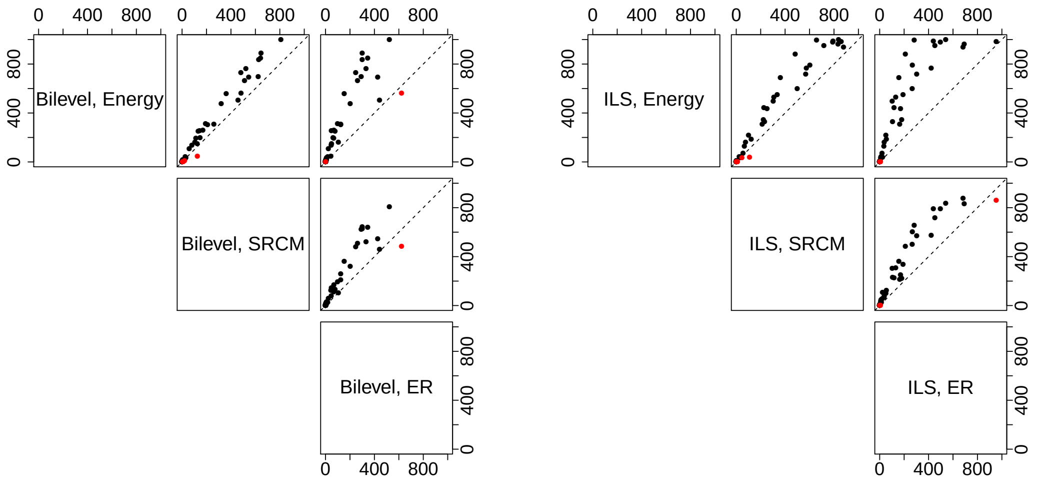

Bilevel protocol (three archivers)		
Mack-Skillings MS Statistic:		41.093
Upper-tail p-value:		1.19×10^{-9}
Critical value of $\lvert S_i - S_j \rvert$:		26.907
Comparison	$S_i - S_j$	Significant?
Energy − SRCM	31.0	Yes
Energy − ER	69.5	Yes
SRCM − ER	38.5	Yes

ILS protocol (three archivers)		
Mack-Skillings MS Statistic:		33.161
Upper-tail p-value:		6.3×10^{-8}
Critical value of $\lvert S_i - S_j \rvert$:		26.907
Comparison	$S_i - S_j$	Significant?
Energy − SRCM	29.0	Yes
Energy − ER	62.5	Yes
SRCM − ER	33.5	Yes

Figure S3: Comparison of three archivers in terms of number of near-native structures in the final decoy set following stage 3. The energy-based and SRCM archivers typically retain larger numbers of native-like decoys than the ER archiver, and the energy-based archiver typically outperforms SRCM. For each protocol, scatterplots compare every pair of archivers in terms of the total number of decoys retained by these archivers (out of a total of 1000) that were within 4 Å Cα RMSD from the native, for all 59 targets following stage 3. Data were collected over 100 runs of the bilevel and ILS protocols. In each scatterplot, a dashed line of unit slope is drawn, and points lying below this line are coloured red. Data for these targets are shown in Table 1. Results of statistical testing using the Mack-Skillings test and the corresponding post-hoc procedures are shown below each set of scatterplots.

Table S1: Numbers of retained structures in each archived set that were within 4 Å Cα RMSD from the native after stage 3 (out of a total of 1000). Data are shown for targets where the SRCM or ER archive retains a greater number of native-like structures than the energy-based archiver, in at least one of the two running protocols. For all other targets, the energy-based archiver retained a greater number of near-native decoys over 100 runs, as compared to the SRCM and ER archivers. Data for all targets is given in Table S5. Boldface values indicate the highest observed value for a given target and protocol.

Target	Bilevel protocol			ILS protocol		
	Energy	SRCM	ER	Energy	SRCM	ER
1a32	45	**126**	44	37	**109**	23
1c9oA	1	**10**	0	2	**9**	1
1cc8A	**25**	**25**	7	34	**46**	11
1ctf	**41**	26	18	0	**2**	0
1elwA	564	483	**623**	**982**	860	954
1ew4A	**13**	5	4	0	0	**1**
1tif	12	**20**	3	**34**	33	9
1ughI	0	0	0	0	**1**	0
1utg	0	**3**	1	0	2	**6**
4ubpA	**19**	17	3	2	**9**	6

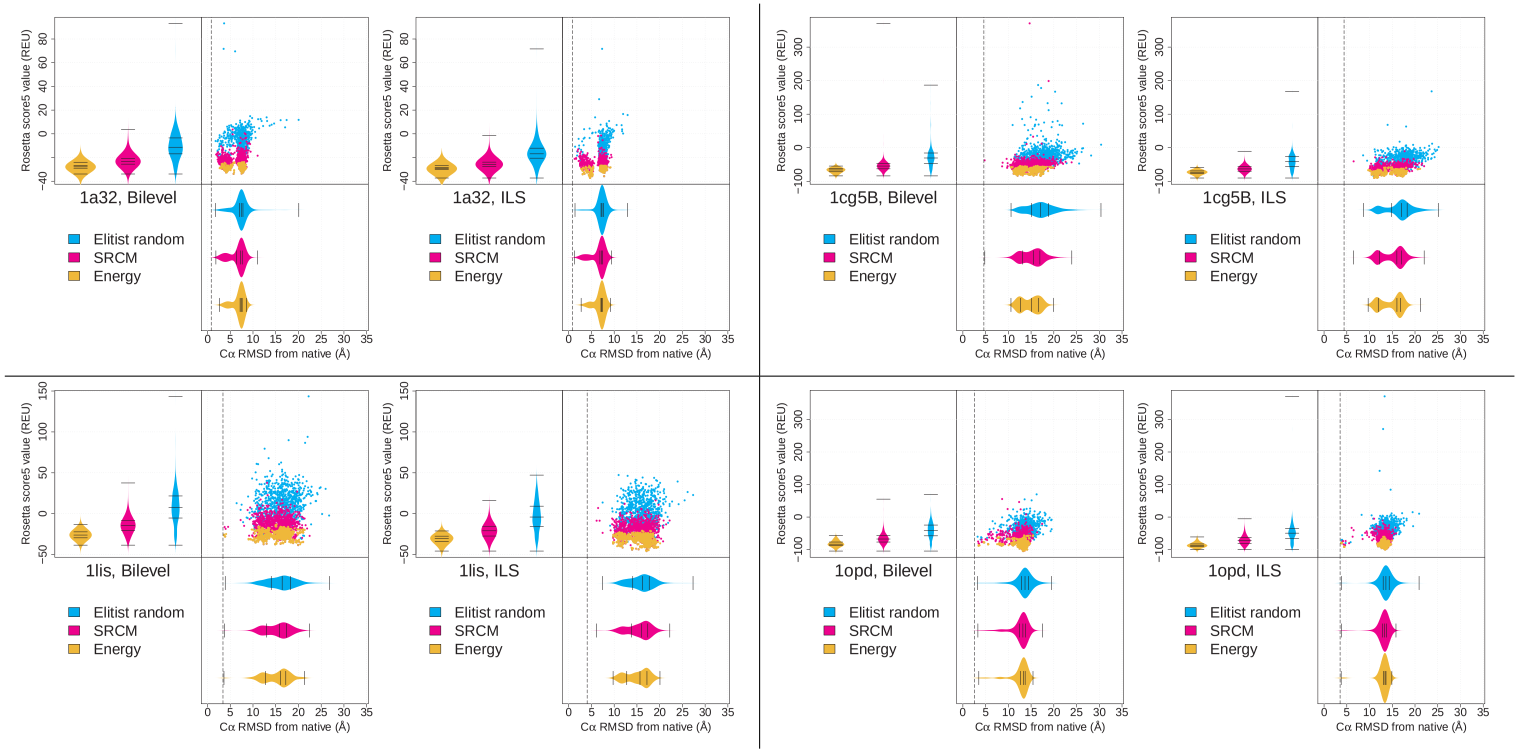

Figure S4: Score versus RMSD data for four additional targets, following stage 3 of our protocols, contrasting successes and failures of the SRCM strategy. For target 1a32 (top left), the SRCM archiver retains an improved number of near-native structures as compared to the other two archivers. For the remaining three targets, such improvements are either rare or absent relative to the other two archivers. Data are represented as in Figure 4 in the main text.

Figure S4 shows the score and RMSD data for four additional targets (1a32, 1lis, 1cg5B and 1opd) following stage 3 of our protocols. As mentioned above, for target 1a32, the SRCM archiver reliably retains an improved number of near-native structures as compared to the other two archivers following stage 3, and we find this is the case for runs of both the bilevel and ILS protocols. For this target, we see that very low values of the RMSD measure tend to associate with higher scores. Crucially, it appears that a large number of such low-RMSD, high-score structures are actually encountered as *LMin*s, despite the fact that the most frequently accessed *LMin*s are farther away from the native. The lowest fraction of native-like *LMin*s accessed in any single run is 0.038 for the bilevel protocol and 0.015 for the ILS protocol, indicating that good *LMin*s are accessed reliably across independent runs. Further, since the most frequently accessed set of states are found at Cα RMSD values of approximately 7 Å from the native structure, this means that native-like states encountered during the search would be quite favourable under the diversity criterion in the SRCM archiver. Since many of these structures have reasonable score values as well, they can be retained frequently.

On a few other targets, we see some evidence of beneficial effects when using the SRCM archiver (and in some cases the ER archiver) in terms of retaining solutions with better RMSD values (e.g. 1cg5B, 1opd and 1lis in Figure 4). For some of these targets, the SRCM and ER archivers do retain a slightly larger number of native-like decoys as compared to the energy-based archiver. We find that in many cases across the whole set of targets, these favourable decoys originate from one or a handful of runs for the target and protocol. For these runs, the retention of native-like solutions correlates with an improved fraction of native-like *LMin*s accessed during the particular run as well. This indicates that solutions of similar quality are simply too difficult to reach reliably, and instead are only accessed in a handful of runs.

S3 Analysis of score values in the three archivers

Considering the distributions of score values obtained by the three archivers, we find that the ER archiver retains structures with systematically higher scores than the other two archivers. The higher values of score seen in the ER archiver's solutions is a consequence of the nature of the search algorithms employed in our protocols. As outlined in Kandathil et al. (2018), our protocols achieve improved conformational exploration relative to Rosetta through the use of schemes of forced perturbation and local search. Further, as mentioned previously, the ER archiver retains a mostly random subset of all the *LMin*s sampled in our methods, meaning that the score values seen for this archiver reflect the overall distributions of scores in all the *LMin*s, to some extent. Due to the potentially drastic nature of the structural change that can be introduced by the perturbation steps in our search algorithms, and the fact that the local search steps are not designed to carry out a detailed optimisation of the perturbed structure, *LMin* structures can have unfavourable score values. The reduced quality in score terms also translates into the ER archiver typically exhibiting a relatively higher number of non-native-like solutions following stage 3. In contrast to the ER archiver, the energy-based archiver

(on account of its retention criteria) retains much lower-scoring structures. This often associates with lower RMSD values as well (Kandathil et al., 2018). Structures retained by the SRCM archiver are in between the energy-based and SRCM archivers, reflecting the fact that in the SRCM archiver, score values are not as heavily favoured during the ensemble reduction procedure as they are in the energy-based archiver. Due to the fact that all three archivers share the use of the elitist step during ensemble reduction (see Methods in main text), all archivers retain the lowest-scoring structure in every run. This leads to identical minimum values of score across the three archived sets of decoys, following stage 3.

S4 Analysis of decoys following stage 4

Most of the discussion in our study was concerned with structures sampled in stages 2 and 3 of our protocols (our sampling algorithms are only active in these stages), and therefore speak only to the accessing and retention of native-like structural states when using 9-residue fragments. We investigated the possibility that following further conformational sampling in stage 4 (which uses 3-residue fragments), decoys from the SRCM and ER archivers may exhibit more favourable RMSD values as compared to the energy-based archiver.

We found evidence of such improvements for a few targets (e.g. 1cg5B, 1a32, 1ig5A). Figure 5 compares pre- and post-stage 4 score and RMSD values for the targets 1a32 and 1cg5B. Comparing the data for target 1cg5B in this figure to that shown in Figure 1 in introduction to the main text, we see that the post-stage 4 distributions of decoys are more comparable, and shows that a small number of decoys showed improved RMSD values following stage 4, although in general these values are still too high to be considered high-accuracy predictions. Complete score and RMSD data for all targets, following stages 3 and 4 of our protocols can be found on Zenodo at `https://zenodo.org/record/3356897`.

Considering the distributions of score values, we see that the strong separation in these distributions between the different archived sets are much less pronounced following the application of stage 4. This is because the nature of the sampling process in stage 4 is different to that employed in stages 2 and 3 in our protocols. Stage 4 in our protocols corresponds to stage 4 of standard Rosetta (Kandathil et al., 2018), which does not employ the scheme of perturbation and local search that our methods employ. As a consequence of this, the archiving procedures are no longer active (there is no equivalent concept of *LMin*s in stage 4), and each archived solution is optimised independently. The differences in the nature of the search protocol in this stage, coupled with the fact that the different archiving strategies are no longer active, leads to differences in the score distributions being less pronounced. Nevertheless, we do observe that typically, the decoy set retained by the ER archiver exhibits a tail of higher score values than the other two archived sets (this can be seen in Figure 5).

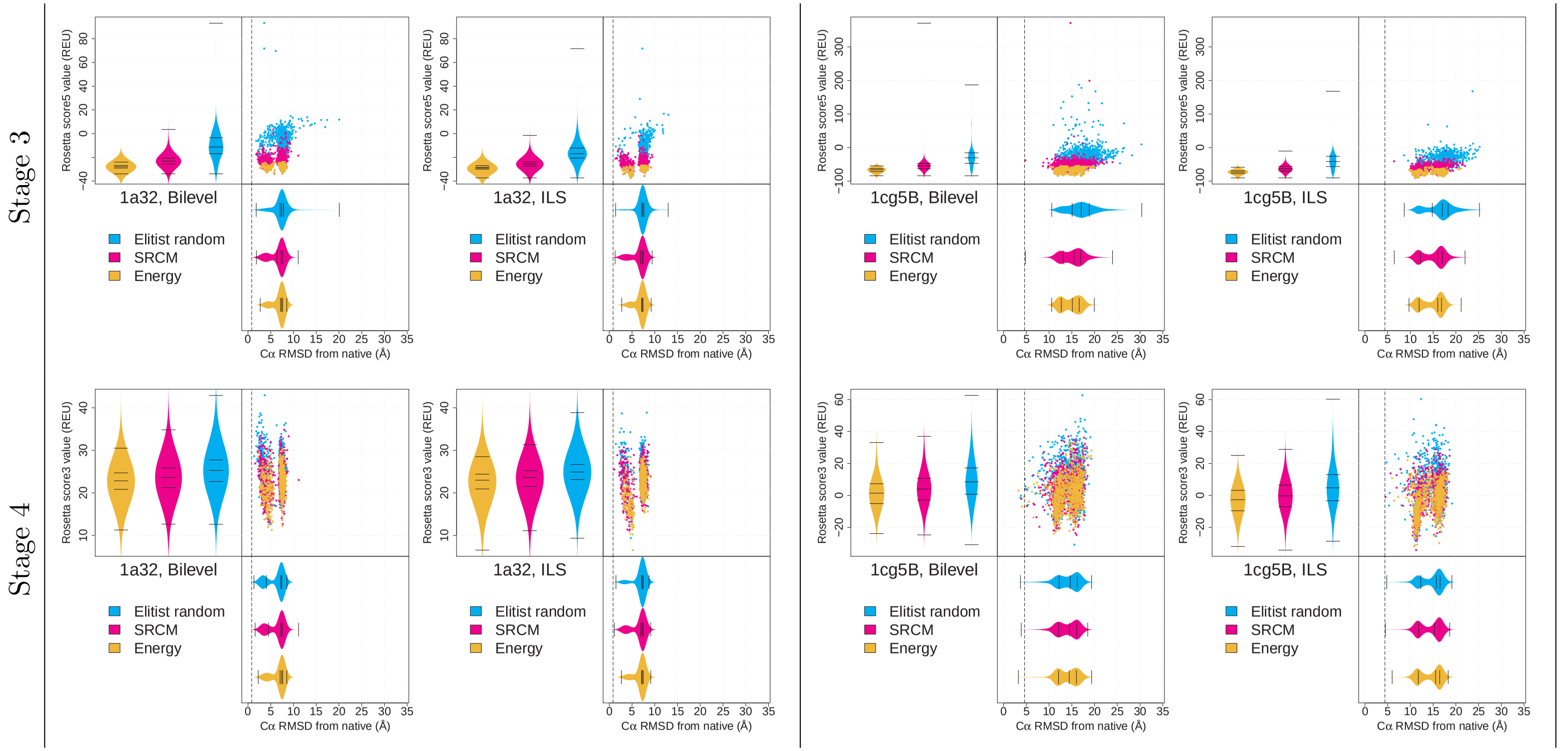

Figure S5: Score versus RMSD data for targets 1a32 and 1cg5B, following stages 3 and 4 (top and bottom row, respectively). For target 1a32 (left column), the optimisation process in stage 4 results in the structures retained by ER archiver showing the greatest number of decoys within 4 Å Cα RMSD from the native, as compared to the SRCM and energy-based archives. Following stage 3 for this target, the SRCM-archived decoy set showed the greatest number of such solutions. For the target 1cg5B, the optimisation process in stage 4 is able to sample a few more structures with improved RMSD values, resulting in a decoy distribution similar to that seen in Figure 1 in the main text. Data are represented as in Figure 4 in the main text.

Following stage 4, we find that the decoy sets retained by the SRCM and ER procedures can occasionally show a higher number of structures within 4 Å of the native. Figure S6 compares the archived sets in terms of the number of decoys within 4 Å Cα RMSD from the native, using similar representations as Figure 3. It can be seen that differences between the energy-based and SRCM decoy sets are no longer statistically significant in either protocol, indicating that the two archived sets present starting points of roughly equal quality for the optimisation procedure in stage 4. Nevertheless, there are some targets for which the SRCM and ER decoy sets exhibit a greater number of native-like solutions (red points in Figure 6). Data for these targets are listed in Table 2. While the SRCM and/or ER decoy sets for these targets do show that improvements in structure quality are possible following the application of stage 4, for most targets, the energy-based archiving strategy still shows the best performance (in terms of the number of decoys within 4 Å of the native). Further, these improvements could be a result of the fact that structures retained by the SRCM and ER archivers tend to be less strongly optimised (have higher energy values; see Figure 4) than those retained on the basis of energy. It is also worth mentioning that the optimisation process in stage 4 is a stochastic process, and is only applied to each decoy once. Some of the improvements seen could therefore be a result of random effects, and further investigation into the exact source of these improvements is desirable.

The results following stage 4 suggest that it may be valuable to investigate extending our search algorithms to stage 4 as well. Since this stage uses 3-residue fragments, this effectively changes not only the structural information available to the search, but also the nature of the move operators. This may provide some opportunities for improvement in predictive accuracy in cases where the 9-residue fragment set does not permit access to native-like states, but the 3-residue fragment set does. The scoring function is also altered, potentially meaning that structural states that were highly favourable in stage 3 may no longer be favoured in stage 4. This is one factor that could enable shifts in RMSD distributions that can sometimes be seen between these stages. In the case of the bilevel protocol, this would change the distribution of move sizes that would be possible relative to stages 2 and 3, since maximum fragment size has now reduced from 9 to 3. There would be interactions with predicted secondary structure as well. These would not apply to the ILS protocol, which always makes moves of length equal to fragment length.

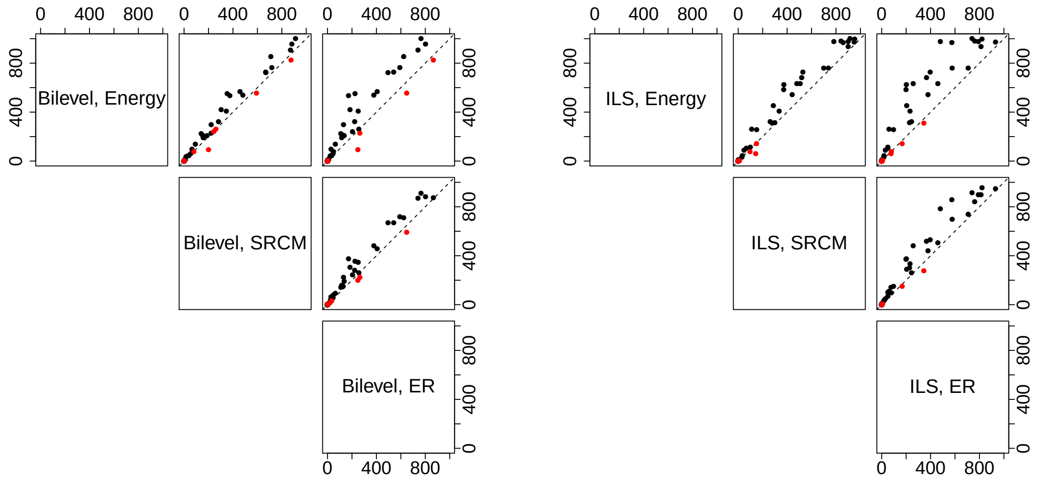

Bilevel protocol (three archivers)

Mack-Skillings MS Statistic:	20.822
Upper-tail p-value:	3.01×10^{-5}
Critical value of $\lvert S_i - S_j \rvert$:	26.907

Comparison	$S_i - S_j$	Significant?
Energy − SRCM	22.5	No
Energy − ER	49.5	Yes
SRCM − ER	27.0	Yes

ILS protocol (three archivers)

Mack-Skillings MS Statistic:	20.093
Upper-tail p-value:	4.33×10^{-5}
Critical value of $\lvert S_i - S_j \rvert$:	26.907

Comparison	$S_i - S_j$	Significant?
Energy − SRCM	20.5	No
Energy − ER	48.5	Yes
SRCM − ER	28.0	Yes

Figure S6: Comparison of three archivers in terms of number of near-native structures in the final decoy set following the application of stage 4 to each archived set. For each protocol, scatterplots compare every pair of archivers in terms of the total number of decoys retained by these archivers that were within 4 Å Cα RMSD from the native, for all 59 targets following stage 4. Each data point represents a target protein, and data were collected over 100 runs of the bilevel and ILS protocols. In each scatterplot, a dashed line of unit slope is drawn, and points lying below this line are coloured red. Data for these targets are shown in Table 2. Results of statistical testing using the Mack-Skillings test and the corresponding post-hoc procedures are shown below each set of scatterplots.

Table S2: Numbers of retained structures in each archived set that were within 4 Å Cα RMSD from the native after stage 4 (out of a total of 1000). Data are shown for targets where the decoy sets archived by SRCM or ER showed a greater number of native-like structures than those retained by the energy-based archive following stage 4, in at least one of the two running protocols. For all other targets, the energy-based archives showed a greater number of native-like decoys over 100 runs, as compared to the SRCM and ER archivers. Data for all targets is given in Table S6. Boldface values indicate the highest observed value for a given target and protocol.

Target	Bilevel protocol			ILS protocol		
	Energy	SRCM	ER	Energy	SRCM	ER
1a32	94	200	**249**	59	**143**	77
1bk2	241	**243**	205	144	150	**168**
1bkrA	0	**1**	0	0	0	0
1bq9A	259	**261**	257	309	278	**346**
1c9oA	**7**	6	**7**	1	**4**	3
1cg5B	1	1	**2**	0	0	0
1ctf	**137**	93	64	3	**11**	3
1enh	230	223	**265**	**543**	442	380
1ew4A	**9**	4	5	0	**1**	**1**
1eyvA	1	**2**	1	**1**	0	**1**
1gvp	0	**1**	0	0	0	0
1ig5A	557	594	**648**	**758**	738	708
1r69	826	**876**	866	**971**	949	932
1tif	**60**	54	46	76	**97**	79
1ughI	0	**3**	1	**9**	6	3
1urnA	75	**80**	49	**114**	99	51
1utg	1	2	**4**	0	1	**4**
2acy	**3**	2	2	0	**1**	0
2vik	0	0	0	0	**1**	**1**
4ubpA	**10**	7	4	6	6	**7**

Table S3: Five-number summaries of the fractions of *LMin*s that were within 4 Å Cα RMSD from the native (Bilevel protocol). Data are taken from 100 runs of the bilevel protocol. 5th percentile RMSD values are also given. RMSD values were calculated using decoys retained by 3 archivers, with 1000 decoys each, following stage 3.

Target	Fraction of *LMin*s within 4 Å of native (n=100 runs)					5th percentile Cα RMSD		
	Min	1st Quartile	Median	3rd Quartile	Max	Energy	SRCM	ER
1a19A	0.0000	0.0001	0.0013	0.0513	0.1466	3.093	3.219	3.561
1a32	0.0382	0.0445	0.0479	0.0536	0.0700	4.112	3.145	4.246
1acf	0.0000	0.0000	0.0000	0.0000	0.0846	4.941	5.668	6.172
1ail	0.0000	0.0000	0.0000	0.0000	0.0002	8.057	7.611	8.363
1aiu	0.1581	0.2421	0.2642	0.2848	0.3535	1.503	1.655	1.740
1b3aA	0.0008	0.0149	0.0308	0.0557	0.1264	3.220	3.162	3.430
1bgf	0.0000	0.0000	0.0000	0.0000	0.0002	10.250	10.418	10.496
1bk2	0.0000	0.0001	0.0008	0.0190	0.1250	3.046	3.324	3.657
1bkrA	0.0000	0.0000	0.0000	0.0000	0.0018	7.111	7.227	8.022
1bm8	0.0000	0.0000	0.0000	0.0000	0.0300	4.347	4.972	5.285
1bq9A	0.0004	0.0027	0.0065	0.0188	0.0710	2.940	3.474	4.169
1c8cA	0.0000	0.0000	0.0000	0.0000	0.0000	7.625	7.773	8.334
1c9oA	0.0000	0.0000	0.0001	0.0008	0.0114	5.021	4.514	5.580
1cc8A	0.0000	0.0001	0.0003	0.0010	0.0263	4.242	4.529	5.055
1cei	0.0000	0.0000	0.0000	0.0000	0.0000	7.486	7.544	8.612
1cg5B	0.0000	0.0000	0.0000	0.0000	0.0000	11.673	11.582	12.210
1ctf	0.0000	0.0000	0.0001	0.0010	0.0515	4.041	4.166	4.397
1dhn	0.0000	0.0000	0.0000	0.0000	0.0000	8.743	8.975	9.893
1elwA	0.3979	0.4816	0.5061	0.5207	0.5594	1.518	1.646	1.633
1enh	0.0037	0.0431	0.0837	0.1253	0.2301	3.489	3.701	3.640
1ew4A	0.0000	0.0000	0.0000	0.0001	0.0241	5.536	5.692	6.213
1eyvA	0.0000	0.0000	0.0000	0.0000	0.0015	5.430	5.764	6.919
1fkb	0.0000	0.0000	0.0000	0.0000	0.0000	13.167	12.766	13.915
1fna	0.0000	0.0002	0.0015	0.0313	0.1421	3.147	3.387	3.786
1gvp	0.0000	0.0000	0.0000	0.0001	0.0016	5.643	5.740	5.956
1hz6A	0.0050	0.1258	0.1477	0.1691	0.2065	2.585	2.671	2.772
1ig5A	0.0014	0.0090	0.0172	0.0385	0.0891	2.834	2.943	3.279
1iibA	0.0000	0.0000	0.0005	0.0064	0.1148	2.980	3.131	3.800
1kpeA	0.0000	0.0000	0.0000	0.0000	0.0000	7.583	7.805	8.451
1lis	0.0000	0.0000	0.0000	0.0000	0.0089	10.706	10.849	11.127
1louA	0.0000	0.0000	0.0000	0.0000	0.0000	6.716	7.074	8.320
1npsA	0.0000	0.0000	0.0001	0.0162	0.0696	3.144	3.438	4.035
1opd	0.0000	0.0000	0.0000	0.0000	0.0417	8.073	8.439	10.553
1pgx	0.0359	0.1051	0.1217	0.1401	0.1755	1.848	1.973	2.657
1ptq	0.0000	0.0000	0.0000	0.0000	0.0000	8.896	8.482	8.904
1r69	0.1608	0.2338	0.2561	0.2866	0.3515	1.803	1.989	2.181
1rnbA	0.0000	0.0000	0.0000	0.0000	0.0000	10.487	11.036	11.881
1scjB	0.0000	0.0456	0.1134	0.1333	0.1751	2.738	2.830	2.990
1shfA	0.0000	0.0001	0.0005	0.0146	0.1329	3.287	3.566	3.820
1ten	0.0000	0.0031	0.0481	0.0721	0.1331	2.515	2.548	3.027
1tif	0.0000	0.0000	0.0001	0.0003	0.0171	4.691	4.611	5.202
1tig	0.0000	0.0000	0.0000	0.0001	0.0606	3.689	3.883	4.524
1tit	0.0000	0.0880	0.1530	0.1875	0.2428	1.763	1.896	2.128
1tul	0.0000	0.0000	0.0000	0.0000	0.0000	9.958	10.204	11.349
1ubi	0.0676	0.1815	0.2047	0.2249	0.2683	2.663	2.466	2.902
1ughI	0.0000	0.0000	0.0000	0.0000	0.0005	6.973	7.590	8.876

continued...

Target	Fraction of *LMins* within 4 Å of native (n=100 runs)					5th percentile Cα RMSD		
	Min	1st Quartile	Median	3rd Quartile	Max	Energy	SRCM	ER
1urnA	0.0000	0.0000	0.0001	0.0003	0.1224	5.479	5.340	6.072
1utg	0.0004	0.0017	0.0026	0.0039	0.0207	8.647	6.220	8.492
1vcc	0.0000	0.0000	0.0007	0.0057	0.0356	4.098	4.179	4.375
1vie	0.0000	0.0000	0.0000	0.0000	0.0001	6.632	6.781	7.650
1who	0.0000	0.0000	0.0000	0.0000	0.0000	8.887	9.417	10.519
1wit	0.0000	0.0000	0.0049	0.0242	0.1169	3.056	3.275	4.025
256bA	0.0096	0.0921	0.1296	0.1576	0.2076	2.278	2.311	2.465
2acy	0.0000	0.0000	0.0000	0.0000	0.0085	7.339	7.904	8.897
2chf	0.0000	0.0216	0.1070	0.1584	0.2546	2.270	2.497	2.624
2ci2I	0.0000	0.0019	0.0457	0.1393	0.2247	2.622	2.747	2.962
2vik	0.0000	0.0000	0.0000	0.0000	0.0000	10.543	10.642	11.625
4ubpA	0.0000	0.0000	0.0000	0.0003	0.0248	4.391	4.999	6.622
5croA	0.2491	0.2972	0.3121	0.3285	0.3984	1.916	2.149	2.003

Table S4: Five-number summaries of the fractions of *LMins* that were within 4 Å Cα RMSD from the native (ILS protocol). Data are taken from 100 runs of the ILS protocol. 5th percentile RMSD values are also given. RMSD values were calculated using decoys retained by 3 archivers, with 1000 decoys each, following stage 3.

Target	Fraction of *LMins* within 4 Å of native (n=100 runs)					5th percentile Cα RMSD		
	Min	1st Quartile	Median	3rd Quartile	Max	Energy	SRCM	ER
1a19A	0.0005	0.0074	0.0245	0.0789	0.1635	3.076	3.267	3.327
1a32	0.0145	0.0213	0.0250	0.0290	0.0532	4.511	3.041	6.649
1acf	0.0000	0.0000	0.0000	0.0000	0.1293	4.229	4.410	4.888
1ail	0.0000	0.0000	0.0000	0.0000	0.0001	6.082	6.143	7.348
1aiu	0.3323	0.3801	0.3965	0.4127	0.4453	1.410	1.492	1.550
1b3aA	0.0040	0.0312	0.0572	0.0855	0.1578	3.110	3.158	3.201
1bgf	0.0000	0.0000	0.0000	0.0000	0.0000	10.260	10.422	10.465
1bk2	0.0001	0.0007	0.0019	0.0067	0.0832	3.211	3.748	4.190
1bkrA	0.0000	0.0000	0.0000	0.0000	0.0005	7.274	7.299	7.556
1bm8	0.0000	0.0000	0.0006	0.0094	0.0734	3.665	3.893	4.167
1bq9A	0.0053	0.0099	0.0162	0.0227	0.0748	3.411	3.514	3.994
1c8cA	0.0000	0.0000	0.0000	0.0000	0.0000	7.869	7.826	8.484
1c9oA	0.0000	0.0000	0.0001	0.0002	0.0019	4.915	4.544	5.680
1cc8A	0.0000	0.0002	0.0004	0.0017	0.0180	4.301	4.059	5.230
1cei	0.0000	0.0000	0.0000	0.0000	0.0001	7.195	7.159	7.430
1cg5B	0.0000	0.0000	0.0000	0.0000	0.0000	11.176	11.206	11.538
1ctf	0.0000	0.0000	0.0000	0.0000	0.0006	5.269	5.185	5.628
1dhn	0.0000	0.0000	0.0000	0.0000	0.0000	8.724	8.999	9.731
1elwA	0.8770	0.8946	0.9012	0.9071	0.9219	1.244	1.329	1.331
1enh	0.0724	0.0929	0.0999	0.1068	0.1279	3.185	3.299	3.511
1ew4A	0.0000	0.0000	0.0001	0.0002	0.0094	6.756	6.572	7.152
1eyvA	0.0000	0.0000	0.0000	0.0000	0.0007	5.611	5.628	5.995
1fkb	0.0000	0.0000	0.0000	0.0000	0.0000	13.414	12.800	14.461
1fna	0.0000	0.0082	0.0520	0.0700	0.1209	2.682	2.895	3.120
1gvp	0.0000	0.0000	0.0001	0.0001	0.0276	5.482	5.780	6.171
1hz6A	0.0738	0.2300	0.2567	0.2738	0.3088	2.494	2.591	2.678
1ig5A	0.0334	0.0707	0.0874	0.1222	0.1809	2.524	2.572	2.790
1iibA	0.0000	0.0006	0.0113	0.0587	0.1291	2.631	2.784	3.290
1kpeA	0.0000	0.0000	0.0000	0.0000	0.0141	6.514	6.608	7.223
1lis	0.0000	0.0000	0.0000	0.0000	0.0000	10.993	11.006	11.517
1louA	0.0000	0.0000	0.0000	0.0000	0.0000	11.793	10.676	11.893
1npsA	0.0000	0.0053	0.0153	0.0379	0.0768	2.649	2.964	3.429
1opd	0.0000	0.0000	0.0000	0.0000	0.0345	12.257	11.692	11.797
1pgx	0.2093	0.2555	0.2725	0.2832	0.3405	1.421	1.520	1.654
1ptq	0.0000	0.0000	0.0000	0.0000	0.0000	8.319	7.681	8.889
1r69	0.4577	0.4945	0.5022	0.5154	0.5378	1.683	1.773	1.791
1rnbA	0.0000	0.0000	0.0000	0.0000	0.0000	10.928	11.435	12.376
1scjB	0.0925	0.1129	0.1206	0.1270	0.1558	2.396	2.628	2.755
1shfA	0.0002	0.0015	0.0124	0.0554	0.1135	2.675	2.889	3.066
1ten	0.0000	0.0604	0.0819	0.0945	0.1174	1.860	2.047	2.239
1tif	0.0005	0.0013	0.0023	0.0048	0.0199	4.097	4.201	4.861
1tig	0.0000	0.0000	0.0001	0.0029	0.1212	3.131	3.443	3.972
1tit	0.0012	0.0986	0.1413	0.1782	0.2500	1.783	1.965	2.116
1tul	0.0000	0.0000	0.0000	0.0000	0.0000	10.800	11.127	12.160
1ubi	0.1917	0.2753	0.2959	0.3099	0.3453	2.569	2.417	2.659
1ughI	0.0000	0.0000	0.0000	0.0000	0.0024	4.660	5.417	6.242

continued...

Target	Fraction of *LMins* within 4 Å of native (n=100 runs)					5th percentile Cα RMSD		
	Min	1st Quartile	Median	3rd Quartile	Max	Energy	SRCM	ER
1urnA	0.0000	0.0002	0.0006	0.0016	0.0414	4.204	4.366	6.114
1utg	0.0094	0.0136	0.0164	0.0188	0.0397	10.619	7.619	5.394
1vcc	0.0004	0.0021	0.0044	0.0126	0.0478	3.947	3.981	4.222
1vie	0.0000	0.0000	0.0000	0.0000	0.0001	8.707	8.063	9.130
1who	0.0000	0.0000	0.0000	0.0000	0.0123	5.916	7.017	9.448
1wit	0.0000	0.0236	0.0526	0.0680	0.1326	2.561	2.753	3.163
256bA	0.0720	0.3460	0.3818	0.4162	0.4795	2.177	2.156	2.227
2acy	0.0000	0.0000	0.0000	0.0000	0.0000	8.803	8.919	9.929
2chf	0.0005	0.0305	0.1674	0.2173	0.2720	2.320	2.516	2.618
2ci2I	0.0000	0.0014	0.0057	0.1701	0.2887	2.470	2.667	2.838
2vik	0.0000	0.0000	0.0000	0.0000	0.0000	8.748	9.182	9.797
4ubpA	0.0000	0.0007	0.0025	0.0064	0.0626	5.623	5.214	5.052
5croA	0.3091	0.3399	0.3536	0.3697	0.4054	1.437	1.591	1.706

Table S5: Numbers of retained structures in each archived set that were within 4Å Cα RMSD from the native after stage 3 (out of a total of 1000). Boldface values indicate the highest observed value for a given target and protocol.

Target	Bilevel protocol			ILS protocol		
	Energy	SRCM	ER	Energy	SRCM	ER
1a19A	**315**	193	99	**437**	253	171
1a32	45	**126**	44	37	**109**	23
1acf	**12**	10	5	**41**	25	14
1ail	0	0	0	0	0	0
1aiu	**1000**	809	523	**1000**	837	540
1b3aA	**306**	209	124	**310**	213	164
1bgf	0	0	0	0	0	0
1bk2	**253**	133	78	**161**	77	37
1bkrA	0	0	0	0	0	0
1bm8	**18**	6	5	**129**	67	35
1bq9A	**139**	81	47	**219**	101	51
1c8cA	0	0	0	0	0	0
1c9oA	1	**10**	0	2	**9**	1
1cc8A	**25**	**25**	7	34	**46**	11
1cei	0	0	0	0	0	0
1cg5B	0	0	0	0	0	0
1ctf	**41**	26	18	0	**2**	0
1dhn	0	0	0	0	0	0
1elwA	**564**	483	623	**982**	860	954
1enh	**160**	105	105	**550**	336	192
1ew4A	**13**	5	4	0	0	**1**
1eyvA	0	0	0	0	0	0
1fkb	0	0	0	0	0	0
1fna	**261**	170	69	**688**	361	157
1gvp	0	0	0	**8**	1	1
1hz6A	**838**	628	301	**979**	790	497
1ig5A	**309**	260	125	**600**	500	266
1iibA	**198**	148	62	**331**	232	106
1kpeA	0	0	0	**6**	1	1
1lis	**9**	3	1	0	0	0
1louA	0	0	0	0	0	0
1npsA	**255**	143	48	**497**	303	103
1opd	**10**	4	4	**10**	2	4
1pgx	**891**	646	301	**990**	792	439
1ptq	0	0	0	0	0	0
1r69	**693**	546	428	**965**	833	692
1rnbA	0	0	0	0	0	0

continued...

Target	Bilevel protocol			ILS protocol		
	Energy	SRCM	ER	Energy	SRCM	ER
1scjB	**730**	481	248	**996**	658	283
1shfA	**195**	113	67	**442**	227	118
1ten	**560**	362	153	**883**	483	210
1tif	12	**20**	3	**34**	33	9
1tig	**108**	59	24	**187**	123	55
1tit	**763**	523	332	**793**	603	268
1tul	0	0	0	0	0	0
1ubi	**848**	642	346	**950**	718	452
1ughI	0	0	0	0	1	0
1urnA	**20**	17	7	**44**	33	12
1utg	0	**3**	1	0	2	**6**
1vcc	**33**	31	9	**72**	56	19
1vie	0	0	0	0	0	0
1who	0	0	0	**10**	2	3
1wit	**149**	124	49	**530**	309	131
256bA	**699**	623	292	**937**	879	683
2acy	**10**	3	1	0	0	0
2chf	**664**	511	262	**718**	569	303
2ci2I	**475**	320	202	**345**	222	180
2vik	0	0	0	0	0	0
4ubpA	**19**	17	3	2	**9**	6
5croA	**506**	459	443	**768**	574	422

Table S6: Numbers of retained structures in each archived set that were within 4Å Cα RMSD from the native after stage 4 (out of a total of 1000). Boldface values indicate the highest observed value for a given target and protocol.

Target	Bilevel protocol			ILS protocol		
	Energy	SRCM	ER	Energy	SRCM	ER
1a19A	**323**	283	223	**634**	507	460
1a32	94	200	**249**	59	**143**	77
1acf	**11**	**11**	8	**42**	34	19
1ail	0	0	0	0	0	0
1aiu	**1000**	912	766	**1000**	916	740
1b3aA	**222**	140	109	**258**	151	98
1bgf	0	0	0	0	0	0
1bk2	241	**243**	205	144	150	**168**
1bkrA	0	1	0	0	0	0
1bm8	**34**	17	21	**88**	48	30
1bq9A	259	**261**	257	309	278	**346**
1c8cA	0	0	0	0	0	0
1c9oA	**7**	6	**7**	1	**4**	3
1cc8A	**43**	40	25	**31**	30	18
1cei	0	0	0	0	0	0
1cg5B	1	1	**2**	0	0	0
1ctf	**137**	93	64	3	**11**	3
1dhn	0	0	0	0	0	0
1elwA	**534**	375	172	**977**	783	481
1enh	230	223	**265**	**543**	442	380
1ew4A	**9**	4	5	0	**1**	**1**
1eyvA	1	**2**	1	**1**	0	**1**
1fkb	0	0	0	0	0	0
1fna	**408**	347	252	**682**	520	368
1gvp	0	**1**	0	0	0	0
1hz6A	**852**	711	624	**980**	842	762
1ig5A	557	594	**648**	**758**	738	708
1iibA	**206**	189	137	**406**	335	232
1kpeA	0	0	0	0	0	0
1lis	**6**	1	0	0	0	0
1louA	0	0	0	0	0	0
1npsA	**298**	223	131	**633**	480	258
1opd	**9**	3	6	**6**	3	2
1pgx	**908**	872	741	**998**	954	822
1ptq	0	0	0	0	0	0
1r69	826	**876**	866	**971**	949	932
1rnbA	0	0	0	0	0	0

continued...

Target	Bilevel protocol			ILS protocol		
	Energy	SRCM	ER	Energy	SRCM	ER
1scjB	**722**	670	496	**969**	859	575
1shfA	**211**	151	129	**455**	288	205
1ten	**420**	304	184	**623**	374	202
1tif	**60**	54	46	76	**97**	79
1tig	**192**	157	117	**312**	300	228
1tit	**551**	353	226	**583**	372	200
1tul	0	0	0	0	0	0
1ubi	**956**	883	803	**978**	900	791
1ughI	0	**3**	1	**9**	6	3
1urnA	75	**80**	49	**114**	99	51
1utg	1	2	**4**	0	1	**4**
1vcc	**43**	33	35	**107**	67	51
1vie	0	0	0	0	0	0
1who	0	0	0	**11**	2	3
1wit	**96**	65	29	**259**	110	61
256bA	**762**	719	592	**937**	900	814
2acy	**3**	2	2	0	**1**	0
2chf	**726**	669	542	**760**	700	577
2ci2I	**567**	458	407	**324**	262	245
2vik	0	0	0	0	**1**	**1**
4ubpA	**10**	7	4	6	6	**7**
5croA	**537**	481	379	**727**	529	397

References

Kandathil, S. M., Garza-Fabre, M., Handl, J., and Lovell, S. C. (2018). Improved fragment-based protein structure prediction by redesign of search heuristics. *Scientific Reports*, 8(1):13694.
